# Experiences and needs of individuals living with diabetic peripheral neuropathy: a qualitative systematic review and meta-synthesis

**DOI:** 10.3389/fneur.2026.1746503

**Published:** 2026-03-09

**Authors:** Xiaohong Xu, Shini Huang, Fang Wang, Liuxue Guo, Shunqi Liao, Maoyi Yang, Zhi Wang

**Affiliations:** 1 College of Nursing, Chengdu University of Traditional Chinese Medicine, Chengdu, China; 2 Guang'an Hospital of Traditional Chinese Medicine, Guang'an, China; 3 Hospital of Chengdu University of Traditional Chinese Medicine, Chengdu, China

**Keywords:** diabetic complications, diabetic peripheral neuropathy, illness experiences, qualitative research, self-management

## Abstract

**Objective:**

This study synthesises qualitative literature on the lived experiences of patients with diabetic peripheral neuropathy (DPN) during their illness, aiming to help healthcare professionals understand patients’ emotional perspectives and perceived needs, thereby facilitating the development of more effective medical interventions.

**Methods:**

The Joanna Briggs Institute meta-synthesis method was used to analyse the 10 included qualitative studies.

**Results:**

By synthesising the original studies, four themes and 11 subthemes were extracted, comprehensively analysing patients’ symptom experiences, physical-psychological-social impacts, self-management strategies, and needs.

**Conclusion:**

Healthcare professionals should emphasise foot care education, understand the connotations of self-compassion and pain catastrophizing, and develop individualised interventions for different populations to enhance patients’ self-care abilities and improve their wellbeing.

**Systematic review registration:**

https://www.crd.york.ac.uk/PROSPERO/view/CRD420251051721, Unique Identifier: CRD420251051721.

## 1 Introduction

With the global prevalence continuing to rise, diabetes mellitus (DM) has become a significant public health issue (1). Surveys show that around 460 million people across the globe had DM diagnoses in 2019, accounting for 9.3% of the world’s population; projections suggest this number will surge to 700 million (10.9%) by 2045 (2). Through its associated complications, DM poses direct threats to patients’ health and survival while imposing substantial socioeconomic burdens, with annual global healthcare costs related to DM exceeding $966 billion (3). Among DM complications, diabetic peripheral neuropathy (DPN) has a high incidence, affecting up to 50% of patients living with DM (4). DPN’s prevalence grows with increasing age and diabetes duration. Among adult patients with type 1 diabetes mellitus (T1DM), the baseline prevalence jumps from 6 to 30% over 13 years (5). In adults with type 2 diabetes mellitus (T2DM), it is even higher, ranging between 39 and 51% (6).

DPN encompasses a wide range of manifestations involving sensory, motor, and autonomic nerve functions, with sensory and motor dysfunction of the distal extremities being key clinical features that disrupt daily life. Additionally, DPN frequently induces neuropathic pain (7), which ranks among the most debilitating symptoms experienced by patients (8). Approximately 15–25% of individuals diagnosed with DPN are affected by this condition (9). Research has demonstrated that patients with DPN exhibit foot biomechanical abnormalities, which, together with neuropathy, increase the risks for foot ulcers (10). Furthermore, further progression of these ulcers often leads to non-traumatic lower extremity amputation (NLEA), with diabetic neuropathy being associated with 50–75% of NLEA cases (11, 12). Notably, DM itself is a major risk factor for peripheral arterial disease (PAD), and the comorbidity rate between the two is high—nearly 50% of DM patients with foot ulcers also suffer from PAD and circulatory disorders (13). This comorbid state exerts a synergistic harmful effect with DPN: building on the increased ulcer risk induced by DPN, it further exacerbates ischemic pain, delays wound healing, and elevates the risks of disability, gangrene, and NLEA (14). For instance, in Canada, PAD and DM together account for 80% of lower extremity amputations, which not only augments patient suffering but also imposes a burden on the healthcare system (15). There exists a bidirectional relationship between symptomatology and psychological states in patients with DPN; this interaction is particularly pronounced in those with comorbid neuropathic pain. Such individuals often experience more severe functional impairments and physical limitations (16), alongside complications like insomnia, heightened anxiety, and depression (17). Furthermore, depressive states can amplify pain perception while reducing quality of life (QoL) (18). Compared with painless diabetic neuropathy, painful neuropathy exerts a notably more severe adverse impact on patients’ psychological wellbeing (19), further contributing to declines in health-related QoL and impaired social interactions. Inadequate pain management can also reduce work efficiency, potentially leading to unemployment or early retirement (20, 21). Notably, painless DPN is also problematic; patients tend to underestimate the severity of painless manifestations, which may lead to delayed medical treatment and indirectly elevate the risk of complication progression (9). The physical and psychological consequences of DPN, combined with restricted social engagement, impose substantial economic burdens on both patients and healthcare systems (6). This burden not only directly increases medical expenses but also reduces work capacity due to neuropathy-related disabilities, resulting in higher indirect costs from lost productivity (22). Studies have confirmed that strict self-management is a key approach to preventing the progression of DM complications (23). Patients with strong self-management capabilities proactively avoid unhealthy behaviours, thereby slowing disease progression, improving health-related QoL, and enhancing mental wellbeing (24). However, Gao et al. (25) demonstrated that self-management capabilities among patients with DPN are generally poor. This phenomenon may be associated with factors such as symptom-related fatigue, uncertainty about disease progression, limited health literacy, economic burdens, and lack of personalized guidance, which contribute to patients’ difficulty maintaining long-term self-management behaviours (25, 26). Patients’ inability to achieve effective self-management due to these multiple barriers directly impairs the effectiveness of existing treatments, making it challenging to achieve optimal disease control goals even with pharmacological interventions (25).

Given the high prevalence of DPN and its negative outcomes, urgent early screening and targeted management approaches are requisite. Clinically, emphasis should be placed on comprehensive management approaches aimed at alleviating symptoms while addressing relevant risk factors (27). Currently, pharmacological treatments for DPN are typically associated with limited efficacy, adverse reactions, and unfavorable long-term outcomes (28). The National Institute for Health and Care Excellence advocates implementing relevant non-pharmacological treatments for patients with DPN (29). Against this backdrop, psychological interventions, exercise therapy, and spinal cord electrical stimulation have gradually been applied in clinical practice (30). With the growing emphasis on health, attention has expanded beyond DPN treatment to include patients’ physical and psychological experiences during the disease course.

Despite the growing number of intervention studies on DPN, most focus on examining physiological mechanisms and clinical treatments (27), relying on quantitative analysis of outcome measures. This limits the ability to capture patients’ subjective perceptions of symptoms, the influence of cultural backgrounds on management practices, and dynamic coping strategies. Unlike quantitative studies, qualitative research captures patients’ authentic illness experiences and psychological changes through interviews, explores the underlying meanings of phenomena, and enables in-depth exploration of the issue. Meta-synthesis can determine the overall saturation of relevant themes and systematically synthesise existing research evidence, offering comprehensive evidence to support research findings (31).

Systematic searches of existing literature revealed no qualitative meta-synthesis focusing exclusively on DPN; the only relevant qualitative meta-synthesis focuses on diabetic foot ulcers, a distinct diabetes complication. Therefore, this study synthesises existing qualitative studies on the lived experiences, emotional perceptions, and needs of patients with DPN during their illness. From the patient perspective, it analyses their illness experiences, psychological trajectories, management strategies, and personal needs; identifies key priorities for physical and psychological care; and explores the potential impact of symptoms on disease management. It informs the development of healthcare interventions that are both medically effective and patient-acceptable, to enhance the effectiveness of comprehensive management and QoL for patients with DPN.

## 2 Methodology

This research employs the Joanna Briggs Institute (JBI) meta-aggregation approach, with pragmatism as its core philosophical foundation. Drawing on the phenomenological emphasis on capturing individuals’ authentic lived experiences, this approach guides the systematic extraction and synthesis of qualitative findings to ensure the results reflect patients’ subjective perceptions and practical needs. The study is reported in line with the ENTREQ statement guidelines (Supplementary File 1) and the PRISMA 2020 statement (Supplementary File 2), the study protocol is registered on PROSPERO (CRD420251051721).

### 2.1 Search strategy

Using a combination of subject terms and free-text keywords, a systematic search of 11 electronic databases was conducted. These databases, including PubMed, Embase, PsycINFO, SCOPUS, CINAHL Complete, Web of Science, Cochrane Library, CBM, CNKI, VIP, and WANFANG, are used for qualitative studies on the lived experiences, feelings, and perceptions of patients with DPN during their illness. References were also traced to identify additional literature, with the search timeframe from database inception to May 2025. Taking PubMed as an example, the search strategy is provided in Supplementary File 3.

### 2.2 Inclusion criteria

Inclusion and exclusion criteria for this study followed the PICoS principle. Participants (P): patients diagnosed with DPN; Phenomenon of interest (I): real - life experiences, feelings, and perceptions of patients with DPN during their illness; Context (Co): the entire process of treatment and life after DPN diagnosis; Study design (S): qualitative studies or mixed - methods studies reporting qualitative findings, with no restrictions on the types of qualitative research methods. For mixed-methods studies, only the qualitative components were included. We excluded duplicate studies, those lacking full texts, unpublished papers or grey literature, as well as conference abstracts, trial protocols, and reviews. Two members independently screened the literature according to the above requirements, and any disagreements were resolved by a third member.

### 2.3 Literature screening results

**Figure 1:**
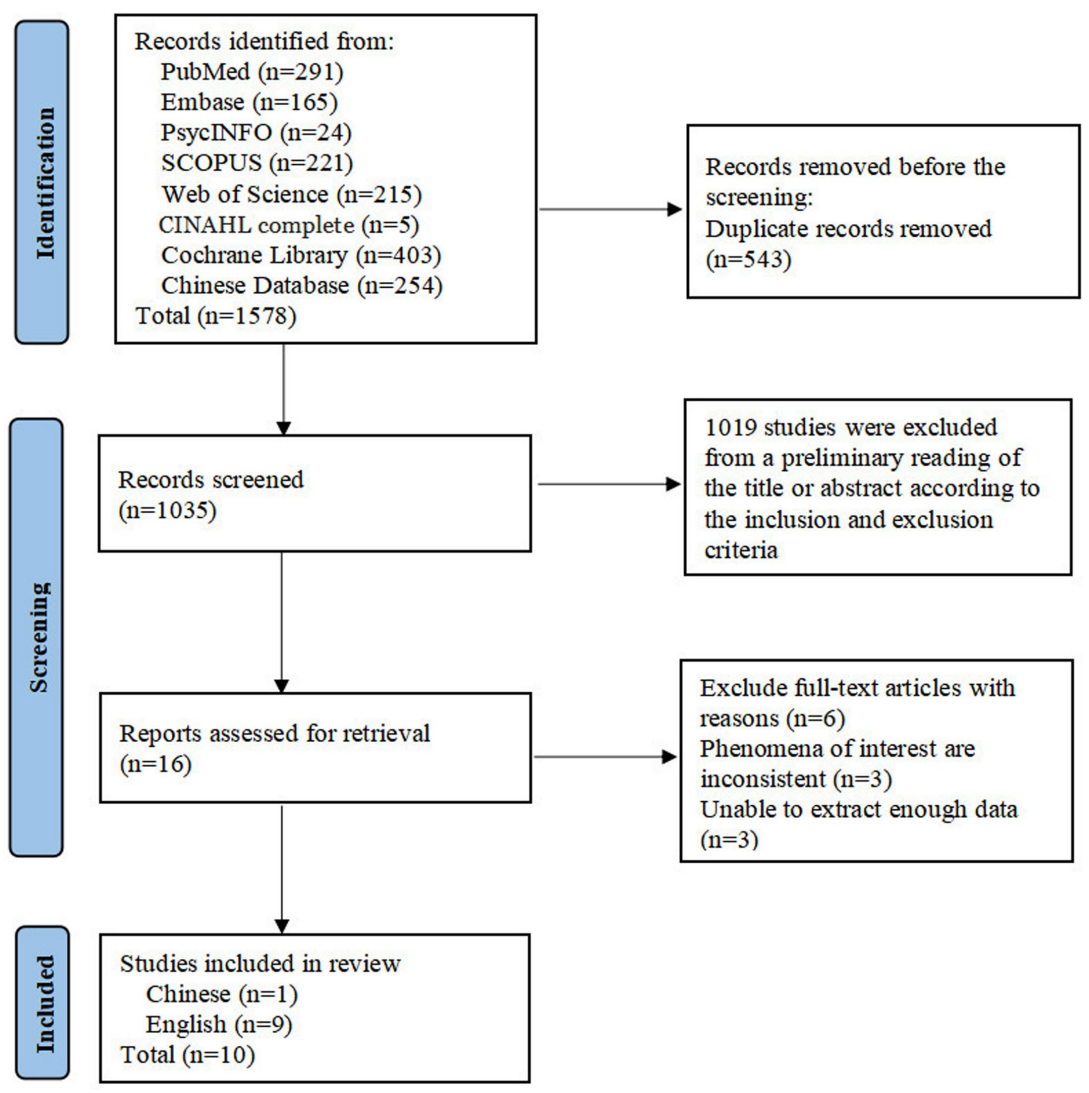
PRISMA flow diagram of literature screening.

The process of literature screening is shown in Figure 1. Through a systematic database search, a total of 1,578 studies were obtained. After removing 543 duplicate studies, 1,019 studies were excluded based on titles and abstracts—with the majority being quantitative studies (n = 671), narrative reviews (n = 83), registered trial protocols (n = 23), or those focusing on biomedical mechanisms (n = 242). Subsequently, a full-text review was conducted on 16 studies. Among them, 3 were inconsistent with the phenomenon of interest in this research, as they focused on caregivers’ experiences, and the other 3 lacked direct participant quotes. This absence of direct quotes failed to capture patients’ authentic subjective feelings and did not meet the data depth requirements for the qualitative synthesis of this study. Eventually, 10 studies were included, comprising 1 in Chinese (32) and 9 in English (16, 33–40).

### 2.4 Quality assessment and data extraction

Two team members independently assessed quality using the JBI qualitative research quality evaluation criteria. Disagreements during assessment were resolved through discussion among all members until consensus was reached. The evaluation covered 10 aspects, each judged as “Yes,” “No,” or “Unclear.” Studies meeting full quality standards with low bias risk were rated A; those partially meeting standards with moderate bias risk were classified as B; and those failing to meet standards with high bias risk were categorised as C. Only studies rated A or B were included.

The quality evaluation results of all included studies are available in Supplementary File 4. All studies were rated as B. Two key methodological deficiencies contributed to this rating. First, none of the studies provided detailed information on the researchers’ own cultural backgrounds, values, or reflexivity. This prevents the full assessment of potential interpretive bias—for example, whether researchers’ cultural perspectives may have influenced the coding or interpretation of participants’ narratives. Second, five of the included studies did not clarify whether there were researcher-related interfering factors, while the remaining five studies did not mention this issue at all. Notably, all studies received ethical approval from the relevant ethics committees, and other evaluation aspects were generally satisfactory.

Two members of the team independently extracted data from the included studies using a standardised form, including study design, characteristics of the study subjects, phenomenon of interest, and main research results.

Table 1 presents the basic characteristics of the included studies. A total of 10 studies were incorporated, with sample sizes ranging from 8 to 99 participants, involving 351 DPN patients in total. Participants’ ages ranged from 26 to 71 years. Of the included studies, seven explicitly recruited participants with painful DPN phenotypes. The remaining three studies only stated enrollment of patients with DPN and did not distinguish between painful and painless DPN subgroups. These studies, published between 2014 and 2024, included four conducted in the United States, with the remainder undertaken across diverse countries: the United Kingdom, China, Denmark, Turkey, Indonesia, and the Netherlands. Four studies employed purposive sampling techniques. All studies used semi-structured interviews, and three studies conducted focus group interviews. Two studies adopted grounded theory, and the remaining studies used phenomenology.

**Table 1:** Characteristics of the included studies.

First author, Year	Country	Study design	Sample characteristics	Age	Phenomenon of interest	Key findings
Brod et al. (36)	USA	Grounded theory Semi-structured interviews focus groups	A total of 70 patients with DPNP^a^, predominantly male (n = 48) Most had T2DM^b^, with only 7 having T1DM^c^	Mean age was 54.0 years (range: 26–70 years)	The burden and impact of pain symptoms on function and well-being among patients with DPNP	1. Physical Function Impacts
						2. Daily-Life Impacts
						3. Social and Psychological Impact
						4. Sleep Impacts
Gok Metin and Arslan, (39)	Turkey	Interpretative phenomenological analysis Semi-structured interviews	A total of 14 patients with DPNP, 8 of whom were female Almost all had T2DM, with only 1 having T1DM The mean duration of diabetes mellitus was 11.07 ± 8.46 years	Mean age: 53.8 ± 10.08 years	The impact of DPNP on daily life and patients’ personal experiences	1. Restrictions of Physical Functions
						2. Difficulties With Daily Routines
						3. Social Limitations
						4. Psychological Impacts
Kanera et al. (16)	the Netherlands	Grounded theory Semi-structured interviews Focus groups	12 patients with PDN^d^, 4 of whom were female All had T2DM and DPN^e^ The mean duration of PDN was 11.3 ± 6.5 years	Mean age: 65.3 ± 10.26 years	Patients with PDN: experiences related to physical exercise and coping strategies	1. Being active despite PDN
						2. Distraction and attention
						3. Avoidance
						4. Acceptance and resignation
Kirk et al. (33)	USA	Phenomenological study Stratified sampling Semi-structured interviews	99 patients with DPN, 63 of whom were female All had T2DM	Mean age: 56.6 ± 10.3 years	The experience of illness among patients with type 2 diabetic neuropathy	1. Patients understood what neuropathy was and attributed their pain and suffering to neuropathy
						2. The pain and suffering caused by neuropathy were nearly insurmountable
						3. Diabetes-related pain led to feelings of frustration and/or hopelessness
Vogel et al. (37)	Denmark	Phenomenological study Purposive sampling Semi-structured interviews	A total of 17 participants, 7 with DPN and 10 with multiple PDN	No relevant information was mentioned in the study	The illness experiences of patients with DPN and multiple PDN, and their impact on quality of life	1. Symptom perception
						2. Impact on daily activities
						3. Impairment of interpersonal relationships
						4. Influence on mental health
Liu et al. (40)	USA	Mixed methods research Semi-structured interviews	A total of 17 patients with PDN, 8 of whom were female. All had T2DM	Mean age: 59.4 years	The perspectives on pain and life experiences of patients with PDN	1. Living with PDN
						2. Quality of life
Davies et al. (38)	UK	Phenomenological study Purposive sampling Semi-structured interviews	A total of 23 patients with PDN, 12 of whom were female 10 had T1DM and 13 had T2DM The mean duration of diabetes mellitus was 23 years (range: 7–50 years)	Mean age: 62 years	Self-management strategies among patients with PDN, and their perspectives on pain management regimens and psychological coping strategies	1. Managing the experience of PDN
						2. Pragmatic approach to pain management
Chun-xiao, (32)	China	Phenomenological studyPurposive sampling Semi-structured interviews Focus group interviews	A total of 80 elderly patients with PDN, including 42 males and 38 females All had T2DM, with a disease duration of 1.19 ± 0.14 years	Mean age: 71.19 ± 2.28 years	The psychological experiences of elderly patients with DPN, as well as their perceptions and expectations regarding exercise rehabilitation	1. Patients’ psychological behaviours
						2. Perceptions of exercise rehabilitation
						3. Expectations toward exercise rehabilitation
						4. Needs for exercise rehabilitation
Storey et al. (34)	USA	Phenomenological study Purposive sampling Semi-structured interviews	Total of 11 breast cancer survivors with DPN	Aged from 41 to 71 years	The illness experiences of breast cancer survivors with DPN	1. Experiences of neuropathic symptoms
						2. Impact on physical function
						3. Impact on quality of life
						4. Management strategies
						5. Influence of diabetes and breast cancer on pain symptoms
Ritonga et al. (35)	Indonesia	Phenomenological study Purposive sampling Semi-structured interviews	A total of 8 patients with T2DM and DPN, 6 of whom were female. The mean duration of T2DM was 6 ± 3.207 years	Mean age: 48.38 ± 13.606 years (range: 27–65 years)	Experiences of T2DM with DPN and associated lifestyle factors	1. Physical activity level
						2. Diet
						3. Sleep pattern
						4. Habit of consuming sweet drinks
						5. Smoking habit
						6. Social interaction
						7. Self-care

^a^DPNP, Diabetic Peripheral Neuropathy Pain; ^b^T2DM, Type 2 Diabetes Mellitus; ^c^T1DM, Type 1 Diabetes Mellitus; ^d^PDN, Painful Diabetic Neuropathy; ^e^DPN, Diabetic Peripheral Neuropathy.

### 2.5 Data synthesis methods and ConQual assessment

This study integrated results using the JBI meta-synthesis approach for evidence-based healthcare. In the first stage, researchers comprehensively reviewed included studies, categorising and merging them based on the similarity of result meanings. Ultimately, new “synthesised findings” were derived from these categories, with comprehensive, representative conclusions for each.

All authors reviewed the data analysis and synthesised results to ensure consistent interpretations and appropriate themes. Two reviewers assessed the credibility and reliability of integrated evidence using the ConQual scoring system. Notably, the ConQual system functions similarly to the GRADE system in quantitative research, as both standardize evidence quality assessment for evidence-based practice—ConQual for qualitative synthesis and GRADE for quantitative studies. Any disagreements during review or assessment were resolved through discussion among all members until a consensus was reached.

Most included studies received a reliability score of 3. However, qualitative studies had clear evidence levels, with no impact on credibility. The ConQual level was ultimately determined as moderate. ConQual system scores are available in Supplementary File 5.

## 3 Results

DPN has multiple adverse effects on patients’ physical functions, psychological states, and daily activities. As the disease progresses, symptoms gradually impair normal physical functions. Pain-related sleep disorders increase fatigue, while persistent declines in energy and attention make daily activities and work difficult to manage. Due to limited mobility and uncontrollable pain, patients abandon hobbies, jobs, and social activities, significantly reducing their QoL. Long-term distress from DPN also harms mental health; patients often experience emotional distress and anxiety about the future. In coping with the disease, patients described their management strategies and unmet needs. The following is a detailed description of this through four themes and 11 sub-themes. The integrated results are shown in Table 2.

**Table 2:** Themes and subthemes.

Themes	Subthemes
Somatic Symptoms and Functional Impairments Caused by the Disease	Symptom perception
	Impairment of physical function
	Sleep disturbances
Multidimensional Impacts on Daily Life	Changes in lifestyle
	Deterioration in quality of life
	Restrictions in social interactions
	Conflicts between role expectations and reality
Impacts on Mental Health	Emotional dysregulation
	Worry about the future
Navigating the Journey of DPN Management	Self-management by patients with DPN
	Management needs of patients with DPN

### 3.1 Somatic symptoms and functional impairments caused by the disease

#### 3.1.1 Symptom perception

Patients described in detail the location, specific sensations, duration, and intensity of perceived symptoms. The most commonly used words to describe symptoms include “numbness,” “tingling,” “pain,” “restlessness,” “burning,” among others. Some patients used analogies to describe their symptoms. For example, they compared pain to sensations such as “It’s like walking on needles” (37). The burning sensation was described as “feel like touching a stove” (34). Some patients even used hand gestures to demonstrate pain location, mimicking sensations such as shooting, stabbing, and rippling pain.

Patients’ descriptions of symptom duration and frequency vary individually, with intensity, duration, and onset time showing individualised patterns. Symptom severity ranges from mild to severe; some patients experience intense pain, described as.

“*Sometimes my feet will hurt really bad and I can’t get up and hardly walk because they’re really hurting*.” (36)

In terms of onset patterns, most patients reported that pain and/or numbness typically worsen at night, while some experience exacerbated symptoms during the day, or describe symptoms as constant, intermittent, or fluctuating. Among them, some patients report persistent symptoms, such as:

“*I live with that every single day, every minute of every day. That’s where my pain is*.” (33)

#### 3.1.2 Impairment of physical function

Patients with DPN experience significant limitations in physical function as the disease progresses, with a more pronounced impact when combined with pain. Common issues include difficulty walking, difficulty standing, impaired balance, reduced mobility, and even decreased self-care ability. Patients cannot walk or stand for long periods:

“*It hurts a heck of a lot to walk around so I generally try not to*.” (36)

Some patients have impaired balance and are prone to falls:

“*I have neuropathy in my hands, my feet and it’s going to my legs and my knees. It’s making me fall*.” (33)

Decreased physical function can reduce patients’ self-care ability. For example, pain or numbness in the hands and fingers can hinder object grasping:

“*It [PN] just made it more challenging for me, even getting dressed, buttoning clothing items*.” (34)

#### 3.1.3 Sleep disturbances

Most patients reported that their sleep has been significantly affected. Neuropathy-induced pain or other uncomfortable sensations impair sleep quality, causing difficulties falling asleep, sleep interruptions, and other disruptions to normal sleep rhythms.

“*very painful at night, preventing sleep*.” (34)

“*At night, it is hard to sleep, at night you only want 3 hours of sleep*…” (35)

Poor-quality sleep can further affect patients’ daytime energy levels, leaving them feeling fatigued and with reduced concentration.

#### 3.2 Multidimensional impacts on daily life

##### 3.2.1 Changes in lifestyle

After being diagnosed with DPN, patients’ lifestyles are more or less affected, which in turn leads to changes. Some patients gradually modified their past habits following a diabetes diagnosis, such as maintaining exercise, altering dietary patterns, limiting sugar intake, and quitting smoking. These habits were integrated into daily management plans for subsequent DPN care.

“*Nothing, after I got diabetes, I never drink sweet tea again*.” (35)

However, some patients continue with “unhealthy” lifestyles, such as not quitting smoking, limiting sugar intake, or controlling their diet. This may relate to insufficient disease understanding and a lack of persistence motivation.

##### 3.2.2 Deterioration in the quality of life

DPN affects patients’ physical function and limits their ability to engage in daily exercise, which is a blow to those who are accustomed to maintaining health through exercise.

“*It has diminished the exercise. It’s hard to*….” (36)

For patients with hand neuropathy, DPN makes it difficult for them to perform fine hand activities, such as dressing, tying shoelaces, doing housework, mowing the lawn, writing, and even using a phone.

“*A little bit of housekeeping, preparing a meal, yes, it costs a lot of effort and pain*…” (16)

Due to difficulty grasping objects, some patients are forced to give up personal hobbies like sewing, writing, painting, cycling, and digital gaming. Additionally, some patients report that DPN negatively affects their sexual relationships, such as diminished desire, erectile difficulties, and avoidance of intimacy with partners due to loss of sensation.

“*I started experiencing reluctance in my sexual life. I want to go to bed immediately. I do not want to be with my husband*…” (39)

“*Well if you’re in pain, you’re not thinking about trying to make love*…” (36)

However, not all studies addressed sexual life-related issues, possibly due to people’s hesitation to discuss this topic.

DPN’s negative impacts extend to the workplace. Discomfort from impaired physical function, combined with disease-related sleep disturbances, significantly depletes patients’ energy and reduces concentration. To adapt to the impacts of the disease, patients may reduce working hours, change jobs, or even resign or retire early.

“*I retired about a year early… I felt that I couldn’t get up and walk around or be as active on my feet as I once was*.” (36)

Inability to fulfil work responsibilities can leave patients feeling frustrated, diminish their sense of personal worth, cause isolation, and even lead to feelings of worthlessness.

“…*I used to be very active at work. Now people pity me because of my disease and do not give me responsibility…I feel useless*.” (39)

DPN’s comprehensive impact on daily activities, hobbies, and work not only alters patients’ original lifestyles but also exerts significant negative effects on their lives, severely reducing QoL.

##### 3.2.3 Restrictions in social interactions

Some patients reported avoiding, concealing, or lying about symptoms, or remaining silent when faced with inquiries from family or partners, to avoid attention or potential conflicts.

“*Sometimes I feel that I have to lie about how I am*…” (37)

Additionally, physical function limitations and pain from the disease prevent patients from participating in outdoor activities for long periods; some passively or actively withdraw from social interactions. This may cause interpersonal problems and feelings of isolation or loneliness.

“*There’s no more socialising, it’s lazy*…” (35)

##### 3.2.4 Conflicts between role expectations and reality

Some studies note a specific phenomenon: some DPN patients experience a mismatch between role expectations and reality. Symptoms may prevent patients from meeting their role expectations, for example, patients who are originally helpful may have to keep others at a distance due to the disease.

“I just have to keep my head up and fight for it. Not show that I’m not always fine. Sometimes, however, I have to stay away from people…” (37)

On the other hand, symptoms can hinder patients from fulfilling their expected social roles. For example, disease-related limitations may prevent them from spending long periods playing with grandchildren, leaving them feeling unfulfilled as grandparents. Or, being unable to continue working due to the disease makes it impossible to fulfil the expected social role.

“*I used to work, my job was construction work, now there are no more activities, just sitting around*.” (35)

Such DPN-related obstacles can threaten patients’ closest relationships and self-perception. As Ribu and Wahl note, patients with DPN fear losing their social roles and responsibilities, thus losing their identity and life goals (41). A widening gap between expected and actual personal states triggers fear of losing one’s identity (42).

### 3.3 Impacts on mental health

#### 3.3.1 Emotional dysregulation

Most patients reported emotional problems triggered by the progression of DPN symptoms, particularly those with comorbid pain. Chronic pain experiences can trigger long-term emotional changes, including worry, irritability, anxiety, hopelessness, frustration, anger, sadness, shame, and feelings of loss or inferiority; some even develop depression.

“…There’s a lot of depression that goes with it because you don’t feel like you want to do anything; you can’t do anything…” (36)

Individual emotional responses vary and relate to inherent personality traits. Undeniably, the disease has brought “endless” troubles, causing emotional fluctuations that are hard to control. They often feel fear and anxiety, and even grow increasingly irritable. Such emotions are uncontrollably directed at others, especially close family members. This undoubtedly causes significant suffering for patients, affects family members directly or indirectly, and even impacts social relationships.

“*I can’t do much of anything because it causes too much pain*…” (34)

“*Somedays you are just irritable and you snap*.” (37)

#### 3.3.2 Worry about the future

In addition to uncontrollable emotions, patients also worry involuntarily about the future. When healthcare professionals inform them of information regarding disease outcomes (such as diabetic foot ulcers), they may experience worry and anxiety, and take certain measures, such as frequent blood glucose monitoring.

“… *I used to test three times a day and now I’m testing five or six times a day*…” (36)

The risk of amputation is a constant concern. They worry that symptoms will worsen over time, eventually leading to amputation or complete loss of independence, and that they will become a burden to their families. This is unacceptable to patients who desire to maintain their independence.

“*When the patient mentioned over 20 possible problems, I felt a bit scared and was upset all day long*.” (32)

“*I can only hope that it won’t get worse than it is now*…” (37)

### 3.4 Navigating the journey of DPN management

#### 3.4.1 Self-management by patients with DPN

In their journey with DPN, patients do not merely receive professional treatment from healthcare professionals; they also seek information through other channels and adopt various symptom management strategies. Over time, they develop confidence in managing the disease, enabling them to face its challenges more resolutely.

“*does not allow the symptoms to impact the quality of life*.” (34)

Pain is the most frequently mentioned troublesome symptom. The common coping strategy is medication for pain relief, sometimes combined with non-pharmacological therapies such as prolonged rest, elevating the affected area, slowing movement, and adjusting footwear or clothing. Some patients attempt pain relief through activities or light exercise, but this is often halted due to intensified pain.

*“Every day I walk 30 min together with my husband. I slowly built.*

*this up.”* (16)

Some patients use alternative coping strategies, such as consuming soothing foods or mental distraction techniques to divert attention and alleviate pain.

“*So, when I get that stuff (neuropathy), I used to be able to take some yogurt or a little dish of cottage cheese or some very hot*…” (33)

Patients adopt different coping methods for specific symptoms. For example, burning sensations may be managed with cold compresses, topical menthol gel, cold water baths, or exposing the feet to cold air. Stabbing pain or pain from light touch may be relieved with hot baths or massages.

“*I had to have the fan on all night on the feet just blowing cold*…” (38)

Some patients may use walking aids to maintain balance and ensure safety to relieve foot pain, numbness, and preserve walking endurance. However, some patients are reluctant to use mobility scooters or wheelchairs even if they have them, as they view this as a sign of “disability”:

“…*Sometimes I have to use a wheelchair, but I try not to, but I sometimes have to resort to that*.” (38)

Patients monitor their condition and actively adopt coping strategies. They engage in regular exercise to sustain physical wellbeing. Although the aims of exercise differ, encompassing the maintenance of strength, fitness, balance, or flexibility. However, some patients use seemingly “incorrect” coping methods, such as fish pedicures, walking through nettles, wrapping feet in plastic wrap, excessive drinking, and marijuana use, though such methods often have little effect.

“…*I’d take my shoes and socks off and I’d walk in stingy nettles tended to take away the other pain temporarily*” (38)

Over time, patients learn to better care for themselves in this process and actively attend regular examinations.

“*I was diligent about going to the health centre, to the hospital*…” (35).

However, not all patients have such conditions. Some note the impact of economic factors; the burdens of poverty, such as financial and housing issues, also hinder disease management.

“*After we pay bills, we have $200 left for two adults and a dog*….” (40)

Beyond relieving physical discomfort, patients adjust their psychological attitudes, such as adopting a positive stance toward the disease and maintaining optimal wellbeing to preserve self-awareness amid DPN.

“…*you know I will dress nicely when you don’t feel like doing it*.” (38)

Such patients actively seek psychological support, including participating in peer support forums, receiving counselling, and communicating with family, friends, or other DPN-experienced patients. Others, however, use negative avoidance strategies, such as avoiding going out.

“*I hate going out*…” (38)

#### 3.4.2 Management needs of patients with DPN

Patients with DPN experience both physical and psychological impacts and urgently need behavioural and psychological support from healthcare professionals. In particular, inpatients may feel uneasy due to the hospital environment and the complexity of their condition:

“*I spend every day with newspapers and books as my companions, and few healthcare providers pay attention to me. It might be loneliness and distress*.” (32)

Compared with information from non-professionals (e.g., family, friends, other DPN patients, and netizens), patients prioritise advice from healthcare professionals. Some patients report being more willing to communicate with professional healthcare professionals than others to obtain accurate information and professional psychological support.

However, DPN management is not always straightforward. Most survey participants are non-professionals and often need guidance from professional healthcare professionals when developing management plans. Patients use individualised coping strategies: alongside doctor-prescribed sleeping pills, they obtain information about therapeutic drugs online, maintain health through independent exercise, and adopt relaxation methods (e.g., listening to music, resting, and distraction) to manage symptom-related sleep disorders. Yet these patients generally report rarely or never receiving professional guidance from healthcare professionals during DPN management, including exercise frequency, recommended and avoidable exercises, sleep-promoting strategies, and psychological support, despite a significant need for such guidance.

## 4 Discussion

This study synthesises qualitative research on the lived experiences, feelings, and perceptions of patients with DPN during their illness. It explores their illness experiences, emotional changes, management strategies, and personal needs from the patient’s perspective, providing reference for developing physical and psychological intervention measures for DPN patients.

### 4.1 Emphasis on foot care education

Daily foot self-care is a crucial self-management objective for patients with DPN (43, 44). Unfortunately, nearly 80% of high - risk diabetic patients have not received foot care education, and up to 60% have never self - inspected their feet or undergone professional foot examinations (45, 46). Therefore, implementing appropriate foot care practices, such as daily foot inspections, regular professional evaluations, maintaining clean and dry feet, avoiding extreme temperatures, and wearing suitable footwear, can significantly reduce the risk of foot complications (44, 47).

Effective foot care education can enhance patients’ awareness of diabetes-related foot issues, improve their foot care behaviours, and reduce the incidence of such problems. Insufficient knowledge is recognised as a major barrier to effective self-care (48). Furthermore, high-quality educational interventions are closely linked to increased knowledge, more standardised self-care practices, and fewer foot complications (49). As an established primary prevention strategy, education fosters behavioural change by empowering individuals to actively engage in self-care (49). Education has been shown to improve patients’ adherence to proper foot care routines, boost their self-efficacy, and reduce the prevalence of foot health risk factors. It also enhances patients’ overall knowledge of diabetes management, as well as their motivation and initiative to adopt healthier behaviours (50–52).

However, educational resources are unevenly distributed. Specifically, patients with visible lesions tend to receive more relevant educational support, whereas low-risk individuals rarely receive information about potential foot complications (53–55). This imbalance in knowledge distribution may lead to detrimental behaviours among some patients and miss critical opportunities for preventing complications (49). Our study found that some patients with DPN experience no obvious discomfort in the early stages. Consequently, they may overlook potential risks, continue their previous lifestyles, or even persist in unhealthy habits. Such behaviour hinders effective DPN management and may increase the risk of developing serious conditions such as foot ulcers. Therefore, for patients with confirmed DPN, early foot care education and evaluation are essential to prevent harmful behaviours that can lead to foot ulcers and amputations.

Notably, our findings demonstrate that the risk of amputation is a core concern for patients with DPN—they frequently worry that if symptoms are not effectively controlled, they will progress gradually to limb loss, subsequently losing their independence in daily life and becoming a burden to their families. This concern holds significant clinical relevance, as a meta-analysis indicated that the overall lower extremity amputation rate in DPN patients reaches as high as 31% (56). Thus, foot care education should explicitly link daily preventive practices (e.g., daily foot inspections, selection of appropriate footwear and socks) to reducing amputation risk. By addressing patients’ core fears and framing foot self-care as a direct safeguard against their most feared outcome, healthcare professionals can enhance patients’ intrinsic adherence, thereby resolving the key barrier of insufficient persistence in healthy habits identified in previous studies.

While current comprehensive systematic reviews lack sufficient evidence to confirm whether foot care education consistently enhances patients’ knowledge of diabetes-related foot issues and adherence to self-care behaviours, its feasibility and potential effectiveness have gradually garnered clinical support. Ren et al. (57) demonstrated that personalized diabetic foot care education helps prevent foot ulcers and reduce amputation rates in high-risk patients. Suglo et al. (58) further confirmed that family-centered foot self-care education tailored to cultural contexts improves patients’ care knowledge and behavioural adherence, reducing related complications. Combined with our qualitative findings on barriers to and preferences for foot care behaviours, this provides a foundation for optimizing future intervention designs. The effectiveness of foot self-care is influenced by multiple factors, including physical fitness, perceived significance, knowledge, education, social integration, risk profile, and the communication between patients and healthcare providers (59–67). Among these, communication between patients and healthcare providers, along with support from social networks, is regarded as a facilitative factor for foot self-care (49). Notably, relying solely on information from family and friends without professional education may lead to cognitive biases and harmful behaviours. Therefore, encouraging DPN patients to actively participate in self-care education and providing professional information support led by healthcare professionals is highly significant for patients.

Studies have confirmed the importance of group education in enhancing patients’ motivation for behavioural change (51). However, education is more likely to be effective when tailored to individual needs. It may succeed when it takes into account the recipients’ needs, abilities, values, attitudes, and beliefs, along with psychosocial elements influencing self-care and help-seeking behaviours (48, 68). This holds particular significance for older patients, who are more susceptible to associated problems like mobility impairment, restricted social support, heightened comorbidities, and cognitive decline, all impacting their capacity for foot self-care.

According to Pallin et al. (69), the goal of diabetic foot care models is to establish a comprehensive care system based on patients’ needs. Combined with our results, it is also necessary to adopt personalised educational content that is updated dynamically as the disease progresses. For timing, interventions should be initiated early, at DPN diagnosis. Even without obvious symptoms, awareness of potential hazards can be raised through case examples and risk alerts.

In terms of educational methods, evidence supports flexible adaptation to patients’ characteristics. As demonstrated in Tuha et al. (70), one-on-one guidance and family collaborative learning are recommended for older patients, who often face barriers such as cultural beliefs or limited mobility; while intuitive formats like diagrams and videos are advisable for those with lower educational levels. In terms of implementation, as highlighted by Tuha et al. (70) regarding social support mechanisms, establishing mutual-aid groups for experience sharing can enhance motivation for self-care, strengthen doctor-patient communication, and assist healthcare professionals in better understanding patients’ concerns and daily behaviours, making educational programmes more acceptable and feasible.

Throughout this process, ongoing assessment of patients’ knowledge and understanding is needed to determine whether they have sufficient foot self-care skills and motivation. Additionally, patients’ perceptions of their own diabetic foot risk may differ from those of healthcare providers, potentially causing communication barriers. Therefore, analysing DPN patients’ perspectives on foot self-management from the healthcare providers’ standpoint, comparing and narrowing the cognitive differences between the two parties, can provide a basis for formulating personalised intervention strategies.

### 4.2 Enhancing self-compassion

Our study identified that most DPN patients experience psychological impacts to varying degrees. Feelings such as “a sense of disability” caused by limited physical functions, “a sense of powerlessness” and “a sense of incompetence” from being unable to work normally, and “a sense of isolation” and “a sense of loneliness” after being forced to withdraw from social activities. Insufficient social support and inadequate disease cognition further exacerbate these negative emotions: patients lacking family understanding and peer communication experience intensified loneliness and helplessness, while cognitive biases such as underestimation of disease risks and uncertainty about disease progression amplify emotional distress and future worries, ultimately leading to depression, anxiety, anger and other negative affective responses. These emotional experiences are consistently reflected in the narratives of participants in the included studies. For example, one participant stated: *“[I’m] starting to get a lot of mood swings…”* (36). Another participant noted: *“… I feel like I’m going to explode. My mental health is breaking down. I feel like I have multiple personalities, both laughing and crying, I experience all things at the same time and it is a very bad situation.”* (39). From the aforementioned emotional experiences and the synthesized theme of “DPN management strategies”, participants’ acceptance and coping with their own predicaments collectively constitute the core manifestations of self-compassion. Its core characteristics are all reflected in participants’ narratives, manifesting as a tendency to be kind to oneself Self-compassion is characterised as “being touched by and open to one’s suffering, not avoiding or disconnecting from it, [and] having the desire to alleviate suffering and heal oneself with kindness” (71). This correlates with the coping strategies for emotional distress among DPN patients in our study. Its core characteristics are all reflected in participants’ narratives, manifesting as a tendency to be kind to oneself [“*… that I’d rather not even start to do it unless it’s absolutely mandatory, and I usually try to finish what I do.*” (36)], objectively accepting one’s own condition [“…*they will be there the next day so if you do not achieve. Something today there is always tomorrow*.” (38)], recognizing a connection with others [“…nobody understands what I’m going through unless it’s another diabetic or it’s somebody else suffering neuropathy because they are going through the same thing.” (38)], and embracing imperfection as part of the human condition (72).

As a useful emotional regulation approach, self-compassion involves facing suffering directly and responding with kindness, understanding, and a sense of common humanity. This helps transform negative emotions, enables individuals to recognise their current situation, and supports them in persisting with effective actions (72). Studies show that higher self-compassion is associated with less depression and anxiety (72, 73), may reduce isolation when facing problems (74), and is linked to fewer reactions to negative events, greater positive emotions, and higher life satisfaction (74, 75). Individuals with self-compassion exhibit fewer emotional overreactions, stronger emotional coping abilities, clearer emotions, and greater capacity to restore emotional states when facing personal weaknesses and life challenges (72).

Self-compassion may reduce distraction and defensive psychology, helping individuals examine health goals calmly, seek timely medical help, and adhere to treatment, thereby promoting healthy behaviours (76). It is persistently linked to improved self-management behaviours (encompassing physical activity, blood glucose monitoring, and healthcare use), metabolic results (HbA₁c), and psychological wellness (77). A meta-synthesis has ascertained that self-compassion strongly forecasts positive health-related behaviours, like nutritious eating, physical exertion, sleep routines, and proficient stress control (78). Research demonstrates that self-compassion is cultivable (78, 79). Intervention studies targeting the enhancement of self-compassion have shown improvements in pain catastrophizing, perceived pain severity, and pain-associated anxiety among chronic patients (80). While self-compassion interventions demonstrate short-term benefits for patients with DPN, Jerawatana et al. (81) showed that a 12-week online self-compassion program significantly improved glycemic control in adolescents and young adults with type 1 diabetes, with this benefit sustained at 24 weeks. However, the study’s small sample size, focus on a narrow age group, and lack of DPN-specific data limit the generalizability of its findings. Given limited relevant evidence, definitive conclusions about long-term efficacy in the DPN population cannot be drawn, highlighting the need for future targeted research and extended follow-up.

Social support can directly influence self-compassion (82), consistent with findings by Ferrari et al. (77). Drawing on social support theory, a robust social support network can enhance patients’ ability to cope with external environmental changes. Emotional support from relatives and friends may boost patients’ sense of wellbeing, belonging, and self-worth, helping them accept their shortcomings with optimism and tolerance (83). Adequate social resources, on the other hand, can strengthen patients’ disease management capabilities, enabling them to approach difficulties with self-compassion rather than self-blame or denial (82). Social support may also enhance coping abilities: sharing experiences and providing suggestions can help patients optimise disease management (84). In this process, patients are more likely to acquire self-care knowledge and skills, translating these into sustained care behaviours and thus improving their self-care ability. Those with stronger self-care ability tend to participate actively in their treatment and care. Such positive behavioural changes may not only improve their condition but also help them develop a positive life attitude and enhance self-compassion.

Patients with stronger self-care ability exhibit higher self-compassion (82). Given our finding that self-compassion is closely associated with psychological distress and self-management behaviours in patients with diabetic DPN, Healthcare professionals should fully grasp the concept of self-compassion and assess patients’ self-compassion levels. For those with low self-compassion, tailored interventions should be developed based on individual characteristics. A mindful self-compassion intervention program can be implemented, which cultivates the cognitive, behavioural, and physical capacities to soothe and comfort oneself during distress through formal meditation and self-compassion practices (79). In addition, for individuals with limited mobility or inadequate access to healthcare resources, the self-compassion-based family empowerment therapy proposed by Rahmani et al. (85) can be adopted to improve relevant health outcomes. These recommendations are grounded in clinical practice and the real needs and experiences of study participants, demonstrating strong feasibility.

therefore, understand the concept of self-compassion and assess patients’ self-compassion levels. For those with lower levels, interventions such as mindfulness self-compassion training, self-compassion writing combined with meditation, mindfulness practice alongside acceptance and commitment therapy, and compassion-centred comprehensive approaches may be implemented. Additionally, organising family or peer support group activities and establishing healthcare professionals-patient social platforms can provide psychological and social support. This can enhance patients’ self-care abilities and, in turn, improve their self-compassion levels.

### 4.3 Reducing pain catastrophizing

Most patients in our study reported pain symptoms. Studies indicate that approximately 30% of people with diabetes develop painful DPN, with neuropathic pain often being the primary symptom prompting them to seek medical attention (86). Among patients, those with T2DM exhibit a higher prevalence of clinical neuropathy than do those with T1DM (87). After adjusting for relevant risk factors, the incidence of pain in T2DM patients remains twice that in T1DM patients (87). Additionally, higher levels of neuropathic pain are reported in T2DM patients, women, and people of South Asian ethnicity (86); female sex is a potential risk factor for painful DPN (88). Psychosocial aspects such as sociocultural variances in gender roles, the extent of pain catastrophizing, and divergent coping strategies might be influencing factors of this phenomenon (89, 90).

From the core manifestations under the synthesized theme of “impacts on mental health” in this study, such as “worry about the future” and “emotional dysregulation,” a dimension of pain-related cognitive and emotional responses—pain catastrophizing—can be extracted. Pain catastrophizing is defined as an individual’s exaggerated cognitive and emotional responses to anticipated or actual pain experiences (91). It is characterised by magnifying the potential adverse effects of pain, struggling to dismiss thoughts of pain, and a sense of helplessness when managing pain (92). This construct is clearly reflected in the narratives of participants in the current study, one participant mentioned “I feel nervous because you don’t know basically what the future holds, if it gets worse, is this pain going to get more intense…” (36), which reflects excessive worry and negative anticipation of pain progression. Another participant stated “When I see people who have lost their hands and feet in the hospital because of the disease, I feel severe anxiety when I have so much pain and fear losing my extremities due to this pain and being a burden on my family.” (39), embodying the catastrophic interpretation of pain-related outcomes and feelings of helplessness.

Studies confirm that pain catastrophizing has both stability and dynamism (93–95). It can predict more severe emotional distress, physical dysfunction, and occupational interference, and is closely linked to pain intensity and depression (93–95). If it is non-threatening, it may lead patients to continue daily activities. Conversely, when pain is misinterpreted as a threat, it can set off a vicious cycle of catastrophic thinking. This leads to excessive dread of pain or injury, which then results in avoiding physical activity, disuse, depression, and disability (96, 97).

Investigations into patients presenting with assorted pain symptoms demonstrate that pain catastrophizing adversely affects depression and anxiety (98, 99). Moreover, it relates to pain-related disability and behaviours. Patients who catastrophize about pain may similarly catastrophize the impairment of their physical activity capacity, further augmenting their sense of disability and reducing QoL (100, 101). Individuals who engage in pain catastrophizing are more inclined to persist with ineffective approaches to coping with pain (102). Focusing on potential pain signals and dangers can result in a more rigid approach to pain management, ultimately reducing coping effectiveness (96, 103). Pain catastrophizing increases pain-related persistent vigilance and fear, and predicts that patients with recurrent pain will avoid key daily activities (104).

This study identified similar phenomena. Some patients described pain as “it would almost make you scream” (34). Due to unbearable pain and fear of pain, they voluntarily gave up daily activities such as socialising, going out, and working, further strengthening their “sense of disability” and leading to social withdrawal, emotional depression, and reduced QoL. Notably, pain catastrophizing is associated with the subjective perception of pain-related loss of physical activity, but not with actual activity levels (16). It is an independent risk factor for emotional distress in DPN patients and is linked to increased disability and decreased physical activity capacity (105). Over 40% of patients with diabetic neuropathy experience emotional distress, with pain catastrophizing being a strong predictor of anxiety and depression (106).

However, it is important to note that the occurrence and impact of pain catastrophizing are influenced by multiple factors, supported by relevant studies. Suso-Ribera et al. (107) found that pain intensity modulates the relationship between pain catastrophizing and physical function in patients with chronic pain. Consistent with participants’ narratives, patients with more severe pain typically report more frequent catastrophic thoughts. Fang et al. (108) further noted that family function is an upstream factor influencing pain catastrophizing in patients with neuropathic pain—greater family support is associated with lower levels of catastrophic thinking, while limited support is linked to more severe catastrophic thoughts. This highlights the complexity of pain catastrophizing in patients with DPN, requiring not only targeting catastrophic thoughts themselves but also considering upstream influencing factors and adaptive coping strategies.

We found that a subset of DPN patients gradually developed active pain coping behaviours in the process of long-term disease management—a manifestation derived from the “Self-management by patients with DPN” under the synthesized theme of “Navigating the Journey of DPN Management” and “Changes in lifestyle” within the synthesized theme of “Multidimensional Impacts on Daily Life”, which further gives rise to the dimension of pain acceptance. Characterised by acknowledging one’s pain, letting go of attempts to control it, and learning to lead a more fulfilling life with pain is “pain acceptance” (109). This definition is consistent with the actual experiences of participants in the current study, as specifically reflected in the following narratives, one participant mentioned “*I’ve decided that I’m just going to walk through the pain and do it anyway because I’ve got to exercise…*” (33), reflecting a balance between accepting pain and proactively maintaining health behaviours rather than completely abandoning goals due to pain; another participant stated, “*But I don’t know, my pain is my pain, I don’t see how anyone else can help with it, I’ve had it now for four or five years and I’m coping with it the way I can.*” (38), demonstrating acceptance of the long-term nature of pain and an attitude of developing adaptive coping strategies based on one’s own circumstances; additional participants shared, “adjusted her life around the pain and discomfort,” “does not allow the symptoms to impact the quality of life” (34), exhibiting the trait of proactively adapting to pain.

For patients who embrace an accepting attitude toward pain, reliance on control-oriented or avoidance coping strategies is reduced, thereby freeing up cognitive and emotional resources for more meaningful pursuits (110). Psychological interventions based on acceptance are regarded as promising for improving coping outcomes in chronic pain patients (111). Studies find that higher pain acceptance levels in chronic pain patients are associated with fewer negative emotions, more positive emotions (112). In addition, the higher the level of pain acceptance, the higher the physical function and physical activity levels, and the lower the fear of activity and levels of psychological distress (113, 114), which aligns with the behaviour of participants in the current study of persisting with exercise despite pain.

Although pain acceptance is only moderately negatively correlated with pain catastrophizing (115), individuals with a more accepting attitude toward pain tend to have lower pain catastrophizing levels (116). This may stem from greater psychological flexibility, enabling a broader and more adaptive response when pain intensifies (110). Current studies indicate that pain acceptance may play a significant mediating role in the relationship between chronic pain and function, with acceptance associated with better outcomes (16).

Given that there is currently no fully satisfactory pharmacological treatment for painful DPN, combined non-pharmacological approaches may be considered, including psychological support, physical measures, transcutaneous electrical nerve or muscle stimulation, and acupuncture. Healthcare professionals can help patients improve pain acceptance and reduce pain catastrophizing through pain neuroscience education, mindfulness therapy, acceptance and commitment therapy, and cognitive behavioural therapy (117, 118). Studies indicate that pursuing goals may influence maintaining activity despite pain (119). Therefore, participation in appropriate activities, setting achievable goals, and determination to accomplish personal goals all contribute to DPN management (16).

Consistent with our qualitative findings, recent quantitative evidence further supports the mediating role of psychological state regulation in linking DPN symptom improvement and QoL enhancement. Borbjerg et al. (120) demonstrated that both painful and painless DPN are associated with reduced QoL and poorer mental health, with pain intensity amplifying these adverse impacts, confirming that symptom-related distress exerts indirect effects on QoL through disrupting psychological wellbeing. Geelen et al. (121) further highlighted that anxiety and pain catastrophizing in patients with painful DPN exacerbate disability and QoL decline, underscoring that psychological maladjustment can magnify the functional burden of physical symptoms. These findings align with the participant narratives in our synthesis: patients frequently described how unmanaged pain (e.g., “constant stabbing pain”) triggered emotional distress (e.g., “anxiety about amputation”), leading to social withdrawal and reduced work capacity—ultimately impairing QoL. Collectively, this evidence confirms that psychological state regulation serves as a critical mediator between symptom control and QoL enhancement, emphasizing the need for integrated interventions targeting both physical symptoms and psychological wellbeing in DPN care.

### 4.4 Strengths and limitations

This study strictly followed the JBI meta-synthesis methodology and used JBI-developed qualitative research quality evaluation criteria to assess and synthesise qualitative studies exploring the real experiences, perceptions, and feelings of DPN patients during their illness.

However, this study has certain limitations. First, only 10 articles were included. However, we systematically verified thematic saturation during data synthesis in accordance with the JBI meta-aggregation guidelines and ConQual quality assessment standards. Using a sequential analysis approach, we coded and synthesized findings in the order of study inclusion; no new themes or subthemes emerged from the ninth and tenth studies, confirming that thematic saturation was achieved for the core research question. Nevertheless, the limited number of included studies prevented the identification of more nuanced or context-specific subthemes.

Second, the included studies were conducted predominantly in Europe, North America, and Asia, with underrepresentation from low-income countries and regions with distinct cultural backgrounds. This further reflects the relative scarcity of qualitative research exploring the subjective experiences of patients with DPN in these areas. Differences in economic levels, cultural factors, and healthcare systems may influence patients’ illness experiences and self-management behaviours, factors that were not fully captured in our synthesis. Consequently, the current findings may not fully reflect the context-specific needs of patients in these underrepresented regions, limiting the generalizability of the results.

Additionally, the methodological rigor of the included studies was assessed as moderate, with no high-quality studies identified, indicating a certain level of potential bias. A key methodological limitation contributing to this moderate rating is the lack of transparency regarding researchers’ own cultural backgrounds, values, and reflexivity across all included studies, as well as the absence of clear clarification on researcher-related interfering factors in five of them. These deficiencies may have introduced potential bias into the original studies, indirectly impacting the overall reliability of the synthesized findings in our meta-synthesis.

Our synthesis focuses on the overall experiences and needs of adult DPN patients with a predominance of T2DM. Due to the lack of adolescent participants and limited representation of T1DM patients in the included studies, we were unable to explore the differentiated needs of these subgroups, leading to intervention suggestions that are more generalizable to the overall adult T2DM population rather than specific subgroups. This is a gap in the current qualitative evidence on DPN.

Future research should prioritize high-quality original studies to explore subgroup-specific experiences and DPN-related subthemes. A clear distinction between painful and painless DPN subgroups is warranted to elucidate the heterogeneity in patients’ subjective experiences, which will yield more targeted evidence for clinical practice. Concurrently, expanding the geographic scope to include low- and middle-income countries and conducting cross-cultural investigations will address current gaps in sample diversity, strengthen the generalizability of research findings, and further refine the evidence base for DPN care.

## 5 Conclusion

As the first qualitative meta-synthesis focusing on DPN patients’ lived experiences, this study synthesised qualitative evidence on the real experiences, perceptions, and feelings of DPN patients during their illness. It helps healthcare professionals understand, from the patient’s perspective, the physical and psychological impacts endured and health management needs, which can be integrated into DPN management strategies.

The results indicate that DPN patients are affected by multiple biopsychosocial impacts of the disease. Analysing patients’ reported self-coping strategies and management needs suggests that healthcare professionals should emphasise foot care education and develop diverse, targeted educational models. Advancing intervention timing, optimising educational formats, and enhancing doctor-patient communication can improve patients’ self-care awareness and practical abilities, thereby reducing the risk of foot complications. Establishing a healthcare professionals-patient communication platform, providing professional guidance and support, and organising family and peer support group activities can enhance patients’ social support and self-compassion. Additionally, combining pharmacological and non-pharmacological therapies may help patients better cope with DPN-related pain, improve pain acceptance, reduce pain catastrophizing, and enhance wellbeing.

## Data Availability

The original contributions presented in the study are included in the article/Supplementary material, further inquiries can be directed to the corresponding author.

## References

[1] Standl E Khunti K Hansen TB Schnell O. The global epidemics of diabetes in the 21st century: current situation and perspectives. Eur J Prev Cardiol. (2019) 26:7–14. doi: 10.1177/2047487319881021, 31766915

[2] Saeedi P Petersohn I Salpea P Malanda B Karuranga S Unwin N . Global and regional diabetes prevalence estimates for 2019 and projections for 2030 and 2045: results from the international diabetes federation diabetes atlas, 9(th) edition. Diabetes Res Clin Pract. (2019) 157:107843. doi: 10.1016/j.diabres.2019.107843, 31518657

[3] Elsayed NA Aleppo G Aroda VR Bannuru RR Brown FM Bruemmer D . 2. Classification and diagnosis of diabetes: standards of care in diabetes—2023. Diabetes Care. (2023) 46:S19–40. doi: 10.2337/dc23-S002, 36507649 PMC9810477

[4] Tesfaye S Selvarajah D. Advances in the epidemiology, pathogenesis and management of diabetic peripheral neuropathy. Diabetes Metab Res Rev. (2012) 28:8–14. doi: 10.1002/dmrr.2239 22271716

[5] Martin CL Albers JW Pop-Busui R. Neuropathy and related findings in the diabetes control and complications trial/epidemiology of diabetes interventions and complications study. Diabetes Care. (2014) 37:31–8. doi: 10.2337/dc13-2114, 24356595 PMC3868000

[6] Hicks CW Selvin E. Epidemiology of peripheral neuropathy and lower extremity disease in diabetes. Curr Diab Rep. (2019) 19:86. doi: 10.1007/s11892-019-1212-8, 31456118 PMC6755905

[7] Perveen W Ahsan H Rameen S Fayyaz S Zaif A Paracha MA . Prevalence of peripheral neuropathy, amputation, and quality of life in patients with diabetes mellitus. Sci Rep. (2024) 14:14430. doi: 10.1038/s41598-024-65495-2, 38910161 PMC11194260

[8] Axelsen JL Kirk U Andersen SB Schmidt JJ Gaarde MB Franck CL . Neural networks involved in painful diabetic neuropathy: a systematic review. Scand J Pain. (2025) 25:69. doi: 10.1515/sjpain-2024-0069 40197380

[9] Shillo P Sloan G Greig M Hunt L Selvarajah D Elliott J . Painful and painless diabetic neuropathies: what is the difference? Curr Diab Rep. (2019) 19:32. doi: 10.1007/s11892-019-1150-5, 31065863 PMC6505492

[10] Hazari A Maiya AG Shivashankara KN. Foot kinetic and kinematic profile in type 2 diabetes mellitus with peripheral neuropathy (a hospital-based study from South India). J Am Podiatr Med Assoc. (2019) 109:36–49. doi: 10.7547/17-059, 29389217

[11] Vinik AI Park TS Stansberry KB Pittenger GL. Diabetic neuropathies. Diabetologia. (2000) 43:957–73. doi: 10.1007/s001250051477, 10990072

[12] Vinik AI. Management of neuropathy and foot problems in diabetic patients. Clin Cornerstone. (2003) 5:38–55. doi: 10.1016/S1098-3597(03)90017-2 12800479

[13] Fitridge R Chuter V Mills J Hinchliffe R Azuma N Behrendt CA . The intersocietal IWGDF, ESVS, SVS guidelines on peripheral artery disease in people with diabetes mellitus and a foot ulcer. J Vasc Surg. (2023) 78:1101–31. doi: 10.1016/j.jvs.2023.07.020, 37724985

[14] Prompers L Schaper N Apelqvist J Edmonds M Jude E Mauricio D . Prediction of outcome in individuals with diabetic foot ulcers: focus on the differences between individuals with and without peripheral arterial disease. The EURODIALE study. Diabetologia. (2008) 51:747–55. doi: 10.1007/s00125-008-0940-0, 18297261 PMC2292424

[15] Kayssi A de Mestral C Forbes TL Roche-Nagle G. Predictors of hospital readmissions after lower extremity amputations in Canada. J Vasc Surg. (2016) 63:688–95. doi: 10.1016/j.jvs.2015.09.017, 26610648

[16] Kanera IM van Laake-Geelen CCM Ruijgrok JM Goossens M de Jong JR Verbunt JA . Living with painful diabetic neuropathy: insights from focus groups into fears and coping strategies. Psychol Health. (2019) 34:84–105. doi: 10.1080/08870446.2018.1518526, 30320508

[17] Tölle T Xu X Sadosky AB. Painful diabetic neuropathy: a cross-sectional survey of health state impairment and treatment patterns. J Diabetes Complicat. (2006) 20:26–33. doi: 10.1016/j.jdiacomp.2005.09.007 16389164

[18] Jain R Jain S Raison CL Maletic V. Painful diabetic neuropathy is more than pain alone: examining the role of anxiety and depression as mediators and complicators. Curr Diab Rep. (2011) 11:275–84. doi: 10.1007/s11892-011-0202-2, 21611765

[19] Moghadam FD Rahami Z Ahmadi SA Reisi S Ahmadi SM. Predicting quality of life and self-care behaviours in patients with painful diabetic neuropathy based on psychological factors. Sci Rep. (2025) 15:6431. doi: 10.1038/s41598-025-91254-y, 39984728 PMC11845703

[20] Ang L Mizokami-Stout K Eid SA Elafros M Callaghan B Feldman EL . The conundrum of diabetic neuropathies-past, present, and future. J Diabetes Complicat. (2022) 36:108334. doi: 10.1016/j.jdiacomp.2022.108334, 36306721 PMC10202025

[21] Pop-Busui R Ang L Boulton AJM Feldman EL Marcus RL Mizokami-Stout K . ADA Clinical Compendia Series. Diagnosis and treatment of painful diabetic peripheral neuropathy. Arlington, VA: American Diabetes Association (2022). 35544662

[22] Savelieff MG Elafros MA Viswanathan V Jensen TS Bennett DL Feldman EL. The global and regional burden of diabetic peripheral neuropathy. Nat Rev Neurol. (2025) 21:17–31. doi: 10.1038/s41582-024-01041-y, 39639140 PMC13011988

[23] Adu MD Malabu UH Malau-Aduli AEO Malau-Aduli BS. Enablers and barriers to effective diabetes self-management: a multi-national investigation. PLoS One. (2019) 14:e0217771. doi: 10.1371/journal.pone.0217771, 31166971 PMC6550406

[24] Xu WH Rothman RL Li R Chen Y Xia Q Fang H . Improved self-management skills in Chinese diabetes patients through a comprehensive health literacy strategy: study protocol of a cluster randomized controlled trial. Trials. (2014) 15:498. doi: 10.1186/1745-6215-15-498, 25527255 PMC4307742

[25] Gao Y Yan K Yan X Xi N Gao J Ren H. Correlation between health literacy and health-related quality of life in patients with diabetic peripheral neuropathy: the mediating role of self-management. Nurs Open. (2023) 10:3164–77. doi: 10.1002/nop2.1566, 36572957 PMC10077377

[26] Costa IG Tregunno D Camargo-Plazas P. I cannot afford off-loading boots: perceptions of socioeconomic factors influencing engagement in self-management of diabetic foot ulcer. ANS Adv Nurs Sci. (2020) 43:322–37. doi: 10.1097/ANS.0000000000000328, 32956088

[27] Yang K Wang Y Li YW Chen YG Xing N Lin HB . Progress in the treatment of diabetic peripheral neuropathy. Biomed Pharmacother. (2022) 148:112717. doi: 10.1016/j.biopha.2022.112717, 35193039

[28] Lee C Lai CW Wu GL Yen S Huang CM Liu ZH . Review of nonpharmacological interventions for delaying the effects of cerebral neuropathy caused by diabetes. Front Endocrinol. (2025) 16:1621448. doi: 10.3389/fendo.2025.1621448, 40937409 PMC12420265

[29] NICE. Neuropathic pain – Pharmacological management. Ra'anana: NICE (2013).

[30] Liampas A Rekatsina M Vadalouca A Paladini A Varrassi G Zis P. Non-pharmacological Management of Painful Peripheral Neuropathies: a systematic review. Adv Ther. (2020) 37:4096–106. doi: 10.1007/s12325-020-01462-3, 32809209

[31] Goodman S. The generalizability of discursive research. Qual Res Psychol. (2008) 5:265–75. doi: 10.1080/14780880802465890

[32] Chun-xiao Z. A qualitative study on the cognition and expectation of exercise rehabilitation in elderly patients with diabetes peripheral neuropathy. Chin. Sci. Technol. J. Database Med. Health. (2022) 5:4.

[33] Kirk JK Hunter JC Mihalko SL Danhauer SC Shumaker SA. Perspectives of pain in patients with type 2 diabetes. Expert Rev Endocrinol Metab. (2019) 14:215–9. doi: 10.1080/17446651.2019.1592674, 30884990

[34] Storey S Draucker C Haunert L Von Ah D. The experience of peripheral neuropathy symptoms in breast Cancer survivors with diabetes. Cancer Nurs. (2024) 47:E279–86. doi: 10.1097/NCC.0000000000001253, 37232534

[35] Ritonga SH Decroli E Prahastuti BS Usman E Bachtiar A Yetti H. Lifestyle of type 2 diabetes mellitus patients with peripheral neuropathy: phenomenological study. J Pak Med Assoc. (2024) 74:S13–s7. doi: 10.47391/JPMA.Ind-RINC-04 39221790

[36] Brod M Pohlman B Blum SI Ramasamy A Carson R. Burden of illness of diabetic peripheral neuropathic pain: a qualitative study. Patient. (2015) 8:339–48. doi: 10.1007/s40271-014-0093-9, 25354872

[37] Vogel S Gylfadottir SS Finnerup NB Jensen TS. Diabetic polyneuropathy and neuropathic pain: findings from a qualitative study. Pract Diab. (2020) 37:211–5. doi: 10.1002/pdi.2307

[38] Davies B Cramp F Gauntlett-Gilbert J McCabe CS. Peoples' experiences of painful diabetic neuropathy: are pain management programmes appropriate? Br J Pain. (2021) 15:450–9. doi: 10.1177/2049463721989753, 34840793 PMC8611298

[39] Gok Metin Z Arslan IE. Diabetic peripheral neuropathic pain from the perspective of Turkish patients: a qualitative study. J Transcult Nurs. (2018) 29:514–22. doi: 10.1177/1043659617753044, 29338623

[40] Liu R Santana T Schillinger D Hecht FM Chao MT. "it gave me Hope" experiences of diverse safety net patients in a group acupuncture intervention for painful diabetic neuropathy. Health Equity. (2020) 4:225–31. doi: 10.1089/heq.2020.0004, 32462104 PMC7247034

[41] Ribu L Wahl A. Living with diabetic foot ulcers: a life of fear, restrictions, and pain. Ostomy Wound Manage. (2004) 50:57–67. 15129612

[42] Kindermans HPJ Huijnen IPJ Goossens M Roelofs J Verbunt JA Vlaeyen JWS. "being" in pain: the role of self-discrepancies in the emotional experience and activity patterns of patients with chronic low back pain. Pain. (2011) 152:403–9. doi: 10.1016/j.pain.2010.11.009, 21216100

[43] Bakker K Apelqvist J Schaper NC. Practical guidelines on the management and prevention of the diabetic foot 2011. Diabetes Metab Res Rev. (2012) 28:225–31. doi: 10.1002/dmrr.2253 22271742

[44] Singh N Armstrong DG Lipsky BA. Preventing foot ulcers in patients with diabetes. JAMA. (2005) 293:217–28. doi: 10.1001/jama.293.2.217 15644549

[45] De Berardis G Pellegrini F Franciosi M Belfiglio M Di Nardo B Greenfield S . Physician attitudes toward foot care education and foot examination and their correlation with patient practice. Diabetes Care. (2004) 27:286–7. doi: 10.2337/diacare.27.1.286 14694015

[46] Al Sayah F Soprovich A Qiu W Edwards AL Johnson JA. Diabetic foot disease, self-care and clinical monitoring in adults with type 2 diabetes: the Alberta's caring for diabetes (ABCD) cohort study. Can J Diabetes. (2015) 39:S120–6. doi: 10.1016/j.jcjd.2015.05.006 26243464

[47] Calle-Pascual AL Durán A Benedí A Calvo MI Charro A Diaz JA . Reduction in foot ulcer incidence: relation to compliance with a prophylactic foot care program. Diabetes Care. (2001) 24:405–7. doi: 10.2337/diacare.24.2.405 11213900

[48] Harvey JN Lawson VL. The importance of health belief models in determining self-care behaviour in diabetes. Diabet Med. (2009) 26:5–13. doi: 10.1111/j.1464-5491.2008.02628.x, 19125754

[49] Matricciani L Jones S. Who cares about foot care? Barriers and enablers of foot self-care practices among non-institutionalized older adults diagnosed with diabetes: an integrative review. Diabetes Educ. (2015) 41:106–17. doi: 10.1177/0145721714560441, 25480398

[50] Nguyen TPL Edwards H Do TND Finlayson K. Effectiveness of a theory-based foot care education program (3STEPFUN) in improving foot self-care behaviours and foot risk factors for ulceration in people with type 2 diabetes. Diabetes Res Clin Pract. (2019) 152:29–38. doi: 10.1016/j.diabres.2019.05.003, 31082445

[51] Nemcová J Hlinková E. The efficacy of diabetic foot care education. J Clin Nurs. (2014) 23:877–82. doi: 10.1111/jocn.12290 23875608

[52] Figueira ALG Gomes L Freitas MF Pace A. Perception of social support by individuals with diabetes mellitus and foot ulcers. Acta Paul Enferm. (2012) 25:20–6. doi: 10.1590/S0103-21002012000800004

[53] De Berardis G Pellegrini F Franciosi M Belfiglio M Di Nardo B Greenfield S . Are type 2 diabetic patients offered adequate foot care? The role of physician and patient characteristics. J Diabetes Complicat. (2005) 19:319–27. doi: 10.1016/j.jdiacomp.2005.02.005, 16260348

[54] McInnes A Jeffcoate W Vileikyte L Game F Lucas K Higson N . Foot care education in patients with diabetes at low risk of complications: a consensus statement. Diabet Med. (2011) 28:162–7. doi: 10.1111/j.1464-5491.2010.03206.x, 21219423 PMC3040291

[55] Ortegon MM Redekop WK Niessen LW. Cost-effectiveness of prevention and treatment of the diabetic foot: a Markov analysis. Diabetes Care. (2004) 27:901–7. doi: 10.2337/diacare.27.4.901, 15047646

[56] Luo Y Liu C Li C Jin M Pi L Jin Z. The incidence of lower extremity amputation and its associated risk factors in patients with diabetic foot ulcers: a meta-analysis. Int Wound J. (2024) 21:e14931. doi: 10.1111/iwj.14931, 38972836 PMC11227953

[57] Ren M Yang C Lin DZ Xiao HS Mai LF Guo YC . Effect of intensive nursing education on the prevention of diabetic foot ulceration among patients with high-risk diabetic foot: a follow-up analysis. Diabetes Technol Ther. (2014) 16:576–81. doi: 10.1089/dia.2014.0004, 25004241 PMC4135324

[58] Suglo JN Winkley K Sturt J. Improving foot self-care in people with diabetes in Ghana: a development and feasibility randomised trial of a context appropriate, family-orientated diabetic footcare intervention. PLoS One. (2024) 19:e0302385. doi: 10.1371/journal.pone.0302385, 38718093 PMC11078378

[59] Schmidt S Mayer H Panfil EM. Diabetes foot self-care practices in the German population. J Clin Nurs. (2008) 17:2920–6. doi: 10.1111/j.1365-2702.2008.02352.x, 19012760

[60] Harwell TS Helgerson SD Gohdes D McInerney MJ Roumagoux LP Smilie JG. Foot care practices, services and perceptions of risk among medicare beneficiaries with diabetes at high and low risk for future foot complications. Foot Ankle Int. (2001) 22:734–8. doi: 10.1177/107110070102200909, 11587391

[61] Olson JM Hogan MT Pogach LM Rajan M Raugi GJ Reiber GE. Foot care education and self management behaviours in diverse veterans with diabetes. Patient Prefer Adherence. (2009) 3:45–50. doi: 10.2147/PPA.S4349, 19936144 PMC2778408

[62] Arcury TA Grzywacz JG Ip EH Saldana S Nguyen HT Bell RA . Social integration and diabetes management among rural older adults. J Aging Health. (2012) 24:899–922. doi: 10.1177/0898264312449186, 22764154 PMC3636064

[63] Pollock RD Unwin NC Connolly V. Knowledge and practice of foot care in people with diabetes. Diabetes Res Clin Pract. (2004) 64:117–22. doi: 10.1016/j.diabres.2003.10.014, 15063604

[64] Perrin BM Swerissen H Payne C. The association between foot-care self efficacy beliefs and actual foot-care behaviour in people with peripheral neuropathy: a cross-sectional study. J Foot Ankle Res. (2009) 2:3. doi: 10.1186/1757-1146-2-3, 19192309 PMC2650692

[65] Bell RA Arcury TA Snively BM Smith SL Stafford JM Dohanish R . Diabetes foot self-care practices in a rural triethnic population. Diabetes Educ. (2005) 31:75–83. doi: 10.1177/0145721704272859, 15779248 PMC1613259

[66] Heisler M Cole I Weir D Kerr EA Hayward RA. Does physician communication influence older patients' diabetes self-management and glycemic control? Results from the health and retirement study (HRS). J Gerontol A Biol Sci Med Sci. (2007) 62:1435–42. doi: 10.1093/gerona/62.12.1435, 18166697

[67] Gale L Vedhara K Searle A Kemple T Campbell R. Patients' perspectives on foot complications in type 2 diabetes: a qualitative study. Br J Gen Pract. (2008) 58:555–63. doi: 10.3399/bjgp08X319657, 18682014 PMC2566520

[68] Funnell MM Brown TL Childs BP Haas LB Hosey GM Jensen B . National standards for diabetes self-management education. Diabetes Educator. (2010) 33:599–614. doi: 10.1177/0145721707305880 17684162

[69] Pallin JA Buckley-O'Farrell K Riordan F McGrath N O'Neill K MacLoughlin D . Implementing an integrated diabetic foot care programme in Ireland: podiatrists' experience. BMC Health Serv Res. (2023) 23:1157. doi: 10.1186/s12913-023-10144-z, 37884981 PMC10601248

[70] Tuha A Getie Faris A Andualem A Ahmed MS. Knowledge and practice on diabetic foot self-care and associated factors among diabetic patients at Dessie referral hospital, Northeast Ethiopia: mixed method. Diabetes Metab Syndr Obes. (2021) 14:1203–14. doi: 10.2147/DMSO.S300275, 33762837 PMC7982550

[71] Neff K. Self-compassion: an alternative conceptualization of a healthy attitude toward oneself. Self Identity. (2003) 2:85–101. doi: 10.1080/15298860309032

[72] Neff KD. The development and validation of a scale to measure self-compassion. Self Identity. (2003) 2:223–50. doi: 10.1080/15298860309027

[73] Neff KD Hsieh YP Dejitterat K. Self-compassion, achievement goals, and coping with academic failure. Self Identity. (2005) 4:263–87. doi: 10.1080/13576500444000317

[74] Leary MR Tate EB Adams CE Allen AB Hancock J. Self-compassion and reactions to unpleasant self-relevant events: the implications of treating oneself kindly. J Pers Soc Psychol. (2007) 92:887–904. doi: 10.1037/0022-3514.92.5.887, 17484611

[75] Allen AB Leary MR. Self-compassion, stress, and coping. Soc Personal Psychol Compass. (2010) 4:107–18. doi: 10.1111/j.1751-9004.2009.00246.x, 20686629 PMC2914331

[76] Terry ML Leary MR. Self-compassion, self-regulation, and health. Self Identity. (2011) 10:352–62. doi: 10.1080/15298868.2011.558404

[77] Ferrari M Dal Cin M Steele M. Self-compassion is associated with optimum self-care behaviour, medical outcomes and psychological well-being in a cross-sectional sample of adults with diabetes. Diabet Med. (2017) 34:1546–53. doi: 10.1111/dme.13451, 28799282

[78] Sirois FM Kitner R Hirsch JK. Self-compassion, affect, and health-promoting behaviours. Health Psychol. (2015) 34:661–9. doi: 10.1037/hea0000158, 25243717

[79] Friis AM Johnson MH Cutfield RG Consedine NS. Kindness matters: a randomized controlled trial of a mindful self-compassion intervention improves depression, distress, and HbA1c among patients with diabetes. Diabetes Care. (2016) 39:1963–71. doi: 10.2337/dc16-0416, 27335319

[80] Costa J Pinto-Gouveia J. Acceptance of pain, self-compassion and psychopathology: using the chronic pain acceptance questionnaire to identify patients' subgroups. Clin Psychol Psychother. (2011) 18:292–302. doi: 10.1002/cpp.718, 20806418

[81] Jerawatana R Weinstein B Phattanasri CN Saetung S Sahakitrungruang T Tachanivate P . The effects of self-compassion in adolescents and young adults with type 1 diabetes: a pilot randomized controlled trial. Ann Pediatr Endocrinol Metab. (2025) 30:190–200. doi: 10.6065/apem.2448224.112, 40335093 PMC12415275

[82] WangJunwei W Ma Du H Yu W. The influencing factors and mechanism of self-compassion in young and middle-aged stroke patient. J Nurs Sci. (2025) 40:1–13.

[83] Tough H Siegrist J Fekete C. Social relationships, mental health and wellbeing in physical disability: a systematic review. BMC Public Health. (2017) 17:414. doi: 10.1186/s12889-017-4308-6, 28482878 PMC5422915

[84] Tian Tian HW-h Zhu R-x Shi Y. Status and influencing factors of social participation among elderly community patients with stroke. J Nanchang Univ Med Sci Ed. (2017) 57:5

[85] Rahmani S Mansoobifar M Sirifi MR Ashayeri H Bermas H. Effectiveness of family empowerment therapy based on self-compassion on self-care and glycosylated hemoglobin in female patients with type 2 diabetes mellitus: a randomized controlled clinical trial. Women’s Health Bulletin. (2020) 7:33–42.

[86] Abbott CA Malik RA van Ross ER Kulkarni J Boulton AJ. Prevalence and characteristics of painful diabetic neuropathy in a large community-based diabetic population in the U.K. Diabetes Care. (2011) 34:2220–4. doi: 10.2337/dc11-1108, 21852677 PMC3177727

[87] Conlin PR Greenlee C Schillinger D Lopata A Boltri JM Tracer H . The National Clinical Care Commission report: improving Federal Programs that Impact Diabetes Prevention and care. Ann Intern Med. (2022) 175:594–7. doi: 10.7326/m21-4175, 35157491 PMC9029008

[88] Elliott J Sloan G Stevens L Selvarajah D Cruccu G Gandhi RA . Female sex is a risk factor for painful diabetic peripheral neuropathy: the EURODIAB prospective diabetes complications study. Diabetologia. (2024) 67:190–8. doi: 10.1007/s00125-023-06025-z, 37870649 PMC10709240

[89] Bartley EJ Fillingim RB. Sex differences in pain: a brief review of clinical and experimental findings. Br J Anaesth. (2013) 111:52–8. doi: 10.1093/bja/aet127, 23794645 PMC3690315

[90] Gerdle B Björk J Cöster L Henriksson K Henriksson C Bengtsson A. Prevalence of widespread pain and associations with work status: a population study. BMC Musculoskelet Disord. (2008) 9:102. doi: 10.1186/1471-2474-9-102, 18627605 PMC2488345

[91] Sullivan MJ Thorn B Haythornthwaite JA Keefe F Martin M Bradley LA . Theoretical perspectives on the relation between catastrophizing and pain. Clin J Pain. (2001) 17:52–64. doi: 10.1097/00002508-200103000-00008, 11289089

[92] Sullivan M Bishop SR Pivik J. The pain catastrophizing scale: development and validation. Psychol Assess. (1996) 7:524–32. doi: 10.1037/1040-3590.7.4.524

[93] Sturgeon JA Zautra AJ. State and trait pain catastrophizing and emotional health in rheumatoid arthritis. Ann Behav Med. (2013) 45:69–77. doi: 10.1007/s12160-012-9408-z, 22915012 PMC3547141

[94] Westman AE Boersma K Leppert J Linton SJ. Fear-avoidance beliefs, catastrophizing, and distress: a longitudinal subgroup analysis on patients with musculoskeletal pain. Clin J Pain. (2011) 27:567–77. doi: 10.1097/AJP.0b013e318219ab6c, 21540739

[95] Edwards RR Cahalan C Mensing G Smith M Haythornthwaite JA. Pain, catastrophizing, and depression in the rheumatic diseases. Nat Rev Rheumatol. (2011) 7:216–24. doi: 10.1038/nrrheum.2011.2, 21283147

[96] Crombez G Eccleston C Van Damme S Vlaeyen JW Karoly P. Fear-avoidance model of chronic pain: the next generation. Clin J Pain. (2012) 28:475–83. doi: 10.1097/AJP.0b013e3182385392, 22673479

[97] Leeuw M Goossens ME Linton SJ Crombez G Boersma K Vlaeyen JW. The fear-avoidance model of musculoskeletal pain: current state of scientific evidence. J Behav Med. (2007) 30:77–94. doi: 10.1007/s10865-006-9085-0, 17180640

[98] Turner JA Jensen MP Warms CA Cardenas DD. Catastrophizing is associated with pain intensity, psychological distress, and pain-related disability among individuals with chronic pain after spinal cord injury. Pain. (2002) 98:127–34. doi: 10.1016/S0304-3959(02)00045-3, 12098624

[99] Benbow SJ Wallymahmed ME Macfarlane IA. Diabetic peripheral neuropathy and quality of life. Q J Med. (1998) 91:733–7. doi: 10.1093/qjmed/91.11.733 10024935

[100] Sullivan MJL Lynch ME Clark AJ. Dimensions of catastrophic thinking associated with pain experience and disability in patients with neuropathic pain conditions. Pain. (2005) 113:310–5. doi: 10.1016/j.pain.2004.11.003, 15661438

[101] Turk DC Melzack R. Handbook of pain assessment. London: Guilford Press (2001).

[102] Sturgeon JA Zautra AJ. Psychological resilience, pain catastrophizing, and positive emotions: perspectives on comprehensive modeling of individual pain adaptation. Curr Pain Headache Rep. (2013) 17:317. doi: 10.1007/s11916-012-0317-4, 23338769 PMC9473682

[103] Crombez G Viane I Eccleston C Devulder J Goubert L. Attention to pain and fear of pain in patients with chronic pain. J Behav Med. (2013) 36:371–8. doi: 10.1007/s10865-012-9433-1, 22614260

[104] Vase L Egsgaard LL Nikolajsen L Svensson P Jensen TS Arendt-Nielsen L. Pain catastrophizing and cortical responses in amputees with varying levels of phantom limb pain: a high-density EEG brain-mapping study. Exp Brain Res. (2012) 218:407–17. doi: 10.1007/s00221-012-3027-6, 22349560

[105] Geelen CC Kindermans HP van den Bergh JP Verbunt JA. Perceived physical activity decline as a mediator in the relationship between pain catastrophizing, disability, and quality of life in patients with painful diabetic neuropathy. Pain Pract. (2017) 17:320–8. doi: 10.1111/papr.12449, 27006136

[106] Selvarajah D Cash T Sankar A Thomas L Davies J Cachia E . The contributors of emotional distress in painful diabetic neuropathy. Diab Vasc Dis Res. (2014) 11:218–25. doi: 10.1177/1479164114522135, 24821753

[107] Suso-Ribera C García-Palacios A Botella C Ribera-Canudas MV. Pain catastrophizing and its relationship with health outcomes: does pain intensity matter? Pain Res Manag. (2017) 2017:9762864. doi: 10.1155/2017/9762864 28348506 PMC5350380

[108] Fang Y Liu M Wu M Zhang J Liu M Niu T . Path analysis between family functioning and mental health in people with neuropathic pain: roles of pain intensity, self-perceived burden, pain catastrophizing, and functional status. Pain Manag Nurs. (2024) 25:e287–94. doi: 10.1016/j.pmn.2024.03.014, 38664088

[109] McCracken LM. Learning to live with the pain: acceptance of pain predicts adjustment in persons with chronic pain. Pain. (1998) 74:21–7. doi: 10.1016/s0304-3959(97)00146-2, 9514556

[110] Thompson M McCracken LM. Acceptance and related processes in adjustment to chronic pain. Curr Pain Headache Rep. (2011) 15:144–51. doi: 10.1007/s11916-010-0170-2, 21222244

[111] Wetherell JL Afari N Rutledge T Sorrell JT Stoddard JA Petkus AJ . A randomized, controlled trial of acceptance and commitment therapy and cognitive-behavioural therapy for chronic pain. Pain. (2011) 152:2098–107. doi: 10.1016/j.pain.2011.05.016, 21683527

[112] Cho S McCracken LM Heiby EM Moon DE Lee JH. Pain acceptance-based coping in complex regional pain syndrome type I: daily relations with pain intensity, activity, and mood. J Behav Med. (2013) 36:531–8. doi: 10.1007/s10865-012-9448-7, 22854886

[113] Gyurcsik NC Brawley LR Spink KS Glazebrook KE Anderson TJ. Is level of pain acceptance differentially related to social cognitions and behaviour? The case of active women with arthritis. J Health Psychol. (2011) 16:530–9. doi: 10.1177/1359105310394229, 21224336

[114] Wright MA Wren AA Somers TJ Goetz MC Fras AM Huh BK . Pain acceptance, hope, and optimism: relationships to pain and adjustment in patients with chronic musculoskeletal pain. J Pain. (2011) 12:1155–62. doi: 10.1016/j.jpain.2011.06.002, 21820969

[115] Kratz AL Davis MC Zautra AJ. Pain acceptance moderates the relation between pain and negative affect in female osteoarthritis and fibromyalgia patients. Ann Behav Med. (2007) 33:291–301. doi: 10.1007/BF02879911, 17600456 PMC2593934

[116] Vowles KE McCracken LM Eccleston C. Processes of change in treatment for chronic pain: the contributions of pain, acceptance, and catastrophizing. Eur J Pain. (2007) 11:779–87. doi: 10.1016/j.ejpain.2006.12.007, 17303452

[117] Peng X-y Zd Fu D-j Deng S. Research progress on the acceptance of chronic pain. Anhui Med J. (2025) 46:778–83.

[118] Jiang Q YZ-w Liu M-t Zhu C-y Song K-y Zheng B-x . Progress on pain catastrophizing in patients with neuropathic pain. Chin J Pain Med. (2025) 31:366–70.

[119] Claes N Crombez G Vlaeyen JWS. Pain-avoidance versus reward-seeking: an experimental investigation. Pain. (2015) 156:1449–57. doi: 10.1097/j.pain.0000000000000116, 25775360

[120] Borbjerg MK Wegeberg AM Nikontovic A Mørch CD Arendt-Nielsen L Ejskjaer N . Understanding the impact of diabetic peripheral neuropathy and neuropathic pain on quality of life and mental health in 6,960 people with diabetes. Diabetes Care. (2025) 48:588–95. doi: 10.2337/dc24-2287, 39932781

[121] Geelen CC Smeets R Schmitz S van den Bergh JP Goossens M Verbunt JA. Anxiety affects disability and quality of life in patients with painful diabetic neuropathy. Eur J Pain. (2017) 21:1632–41. doi: 10.1002/ejp.1067, 28656745

