# Supplementary material for: Experiences and needs of individuals living with diabetic peripheral neuropathy: a qualitative systematic review and meta-synthesis
Source: Front Neurol. 2026 Mar 9;17:1746503. doi: 10.3389/fneur.2026.1746503 (PMC13006262; doi:10.3389/fneur.2026.1746503)
Supplement: Supplementary file 1 [file Table_1.DOCX]

**Supplementary file 1: ENTREQ checklist (Enhancing transparency in reporting the synthesis of qualitative research)** ^†^

| **No. Item** | **Guide Questions/Description** | **Reported on Page** |
| --- | --- | --- |
| 1. Aim | This study synthesises qualitative literature on the lived experiences of patients with diabetic peripheral neuropathy (DPN) during their illness. | P1 |
| 2. Synthesis methodology | Identify the synthesis methodology or theoretical framework which underpins the synthesis, and describe the rationale for the choice of methodology (e.g. meta-ethnography, thematic synthesis, critical interpretive synthesis, grounded theory synthesis, realist synthesis, meta-aggregation, meta-study, framework synthesis) | P5 |
| 3. Approach to searching | The search was pre-planned. Comprehensive search strategies were undertaken to seek all available studies. | P5  Supplementary file 2 |
| 4. Inclusion criteria | Inclusion and exclusion criteria for this study followed the PICoS principle. Participants (P): patients diagnosed with DPN; Phenomenon of interest (I): real - life experiences, feelings, and perceptions of patients with DPN during their illness; Context (Co): the entire process of treatment and life after DPN diagnosis; Study design (S): qualitative studies or mixed - methods studies reporting qualitative findings, with no restrictions on the types of qualitative research methods. For mixed - methods studies, only the qualitative components were included.  We excluded duplicate studies, those lacking full texts, unpublished papers or grey literature, as well as conference abstracts, trial protocols, and reviews. Two members independently screened the literature according to the above requirements, and any disagreements were resolved by a third member. | P6 |
| 5. Data sources | A systematic search strategy designed by a medical librarian was devised to identify studies reporting on the relevant experiences of pregnant women in the epidemic. Searches were executed in seven electronic databases: PubMed, Embase, PsycINFO, SCOPUS, CINAHL Complete, Web of Science, Cochrane Library, CBM, CNKI, VIP, and WANFANG. The query used both subject headings and keywords for each concept. A separate searching strategy was designed and optimized for each database. Searching terms that we used in databases are as follows: “Diabetic Neuropathies”, “emotions”, “experience”, “feeling”, “demand”, “psychology”, “Qualitative Research”. Results were limited to journal articles, dissertations, theses written in English, and published before May 2025.. Conference proceedings, editorials, commentaries, abstracts only, newsletters, addresses, and research protocols were excluded manually. Reference lists of all selected articles were independently screened to identify additional studies left out in the initial search. Taking PubMed as an example, the search strategy is provided in Supplementary File 2. | P7-8 |
| 6. Electronic Search strategy | Supplementary file-2 describes the literature search | Supplementary file 2 |
| 7. Study screening methods | Study selection was performed by applying the eligibility criteria in stages following established guidelines for systematic reviews (Lefebvre et al., 2019). Results of database searches were first imported into the reference management software program Endnote X9.3.3. After the removal of duplicates, titles and abstracts were read for the assessment of eligibility. Then full-text documents were screened to identify the studies that best fulfilled the selection criteria. At each stage of study screening, a minimum of two trained reviewers independently read and evaluate the eligibility. Any discrepancies were discussed to reach a consensus by the research team. All references to the included articles were searched for additional potentially relevant studies. | P6-7 |
| 8. Study characteristics | Table 1 presents the characteristics of the included studies (author(s), year of publication, country, study design, characteristics of the study subjects, phenomenon of interest, and main research results). | Table 1 |
| 9. Study selection results | A flow diagram using PRISMA guidelines for reporting of systematic reviews is presented in Figure 1 in reporting of the selection process and results. | Figure 1 |
| 10. Rationale for appraisal | A minimum of two trained reviewers independently evaluated the methodological rigor of the included literature following the Checklist for Qualitative Research (Critical Appraisal tools for use in JBI Systematic Reviews) (Lockwood et al., 2015). Disagreements during assessment were resolved through discussion among all members until consensus was reached. The evaluation covered 10 aspects, each judged as ''Yes'', ''No'', or ''Unclear''. Studies meeting full quality standards with low bias risk were rated A; those partially meeting standards with moderate bias risk were classified as B; and those failing to meet standards with high bias risk were categorised as C. Only studies rated A or B were included. | P7-8 |
| 11. Appraisal items | 2020 version of Checklist for Qualitative Research (Critical Appraisal tools for use in JBI Systematic Reviews) | P8-9 Supplementary file 3 |
| 1. Appraisal   process | The appraisal was conducted independently by two independent reviewers. The two reviewers discussed if consensus was required. When the evaluation results conflicted, the third researcher decided. | P8-9 |
| 13. Appraisal results | Appraisal results are presented in Table 3 | Supplementary file 3 |
| 14. Data extraction | This approach entails extracting findings from each study, then categorizing them through their similarity in meaning. Then, we subjected these categories to further synthesis to generate more comprehensive findings called synthesized findings. A finding is defined as a verbatim extract of the author’s analytical interpretation of the results or data. As a finding is extracted, the level of ‘plausibility’ should be allocated based on the reviewers’ assessment of the degree of fit, or congruency between the data and the accompanying exemplar quote. There are three levels of plausibility. A finding was rated as “unequivocal” if the congruence of the finding and the illustration accompanied was beyond a reasonable doubt; as “credible” if a clear association between them was lacking; as “unsupported” if the findings were not supported by the data. Only unequivocal and credible findings were included. Not supported findings were not presented in the synthesis or the results (Lockwood et al., 2015). | P9-10 |
| 15. Software | Results of database searches were first imported into the reference management software program Endnote X9.3.3. After the removal of duplicates, titles and abstracts were read for the assessment of eligibility. | P6-7 |
| 16. Number of reviewers | A minimum of two trained reviewers | P8 |
| 17. Coding | JBI meta-aggregation did not use the technique of coding | NA^‡^ |
| 18. Study comparison | The process of aggregation involves the synthesis of findings by categorizing them through the similarity in meaning. Then, we subject these categories to a synthesis to generate more comprehensive findings. | P9-10 |
| 19. Derivation of themes | We subjected these categories to further synthesis to generate more comprehensive findings called synthesized findings. Only unequivocal and credible findings were included. Not supported findings were not presented in the synthesis or the results (Lockwood et al., 2015). | P9-10 |
| 20. Quotations | “No of finding file” provides findings and quotations from the primary studies to illustrate themes and constructs, and identify whether the quotations were participant quotations of the author’s interpretation. | P10-20 |
| 21.Synthesis output | Synthesis output is presented inTable 2. | P10-20 Table 2 |

^†^ Reference: Tong A, Flemming K, McInnes E, Oliver SA, Craig J. Enhancing transparency in reporting the synthesis of qualitative research: ENTREQ. BMC Medical Research Methodology 2012, 12:181.

^‡^ NA means 'not applicable'
