# Supplementary material for: Experiences and needs of individuals living with diabetic peripheral neuropathy: a qualitative systematic review and meta-synthesis
Source: Front Neurol. 2026 Mar 9;17:1746503. doi: 10.3389/fneur.2026.1746503 (PMC13006262; doi:10.3389/fneur.2026.1746503)
Supplement: Supplementary file 3 [file Table_3.DOCX]

|  |
| --- |

**Supplementary file 3: The search strategy of the PubMed database.**

|  | Search strategy | Result |
| --- | --- | --- |
| **#1** | "Diabetic Neuropathies" [mh] | 29313 |
| #2 | 'diabetes mellitus with neuropathy'[tiab] OR 'diabetes neuropathy'[tiab] OR 'diabetic mononeuritis'[tiab] OR 'diabetic mononeuropathy'[tiab] OR 'diabetic neuritis'[tiab] OR 'diabetic neuropathies'[tiab] OR 'diabetic peripheral neuropathy'[tiab] OR 'diabetic peripheral polyneuropathy'[tiab] OR 'diabetic polyneuritis'[tiab] OR 'diabetic polyneuropathy'[tiab] OR 'diabetic sensorimotor polyneuropathy'[tiab] OR 'neuropathies in diabetes'[tiab] OR 'neuropathy in diabetes'[tiab] OR 'peripheral diabetic neuropathy'[tiab] OR 'polyneuropathy in diabetes'[tiab] OR 'diabetic neuropathy'[Title/Abstract] | 15887 |
| **#3** | #1 OR #2 | 35370 |
| **#4** | 'emotions'[tiab] OR 'experience'[tiab] OR 'feeling'[tiab] OR 'need'[tiab] OR 'requirement'[tiab] OR 'demand'[tiab] OR 'psychology'[Title/Abstract] | 2654884 |
| #5 | "Qualitative Research" [mh] | 106571 |
| **#6** | 'qualitative'[tiab] OR 'mixed study'[tiab] OR 'mixed research'[tiab] OR 'mixed method'[tiab] OR 'phenomenology*'[tiab] OR 'grounded theory'[tiab] OR 'ethnography*'[tiab] OR 'case stud*'[tiab] OR 'action research'[tiab] OR 'interview*'[tiab] OR 'focus group'[tiab] OR 'observation*'[tiab] OR 'mixed research'[pt] OR 'Qualitative Research'[pt] | 2037162 |
| **#7** | #5 OR #6 | 2044544 |
| **#8** | #3 AND #4 AND #7 | 291 |
| **Note: mh: MeSH; tiab: tittle/abstract; pt: publication type** | |  |
