# Supplementary material for: Experiences and needs of individuals living with diabetic peripheral neuropathy: a qualitative systematic review and meta-synthesis
Source: Front Neurol. 2026 Mar 9;17:1746503. doi: 10.3389/fneur.2026.1746503 (PMC13006262; doi:10.3389/fneur.2026.1746503)
Supplement: Supplementary file 4 [file Table_4.DOCX]

| **Supplementary file 4: Quality appraisal of the included studies.** | | | | | | | | | | |
| --- | --- | --- | --- | --- | --- | --- | --- | --- | --- | --- |
| **Last name of the first author et al. (publication year)** | **Is there congruity between the stated philosophical perspective and the research methodology?** | **Is there congruity between the research methodology and the research question or objectives?** | **Is there congruity between the research methodology and the methods used to collect data?** | **Is there congruity between the research methodology and the representation and analysis of data?** | **Is there congruity between the research methodology and the interpretation of results?** | **Is there a statement locating the researcher culturally or theoretically?** | **Is the influence of the researcher on the research, and vice-versa, addressed?** | **Are participants, and their voices, adequately represented?** | **Is the research ethical according to current criteria or, for recent studies, is there evidence of ethical approval by an appropriate body?** | **Do the conclusions drawn in the research report flow from the analysis, or interpretation, of the data?** |
| Brod et al. (2014) | Y | Y | Y | Y | Y | N | N | Y | Y | Y |
| Gokmetin et al. (2018) | Y | Y | Y | Y | Y | N | U | Y | Y | Y |
| Kanera et al. (2018) | Y | Y | Y | Y | Y | N | U | Y | Y | Y |
| Krik et al. (2019) | Y | Y | Y | Y | Y | N | N | Y | Y | Y |
| Vogel er al. (2020) | Y | Y | Y | Y | Y | N | N | Y | Y | Y |
| Liu et al. (2020) | Y | Y | Y | Y | Y | N | U | Y | Y | Y |
| Davies et al. (2021) | Y | Y | Y | Y | Y | N | U | Y | Y | Y |
| Zhang et al. (2022) | Y | Y | Y | Y | Y | N | N | Y | Y | Y |
| Storey et al. (2024) | Y | Y | Y | Y | Y | N | U | Y | Y | Y |
| Ritonga et al. (2024) | Y | Y | Y | Y | Y | N | N | Y | Y | Y |
| Abbreviations: Y: Yes, N: No, U: Uncertain | | | | | | | | | | |
