# Supplementary material for: Experiences and needs of individuals living with diabetic peripheral neuropathy: a qualitative systematic review and meta-synthesis
Source: Front Neurol. 2026 Mar 9;17:1746503. doi: 10.3389/fneur.2026.1746503 (PMC13006262; doi:10.3389/fneur.2026.1746503)
Supplement: Supplementary file 5 [file Table_5.DOCX]

**Supplementary file 5: Results of ConQual quality assessment.**

| Synthesized findings | Type of research | Dependability | Credibility | ConQual score | Comments |
| --- | --- | --- | --- | --- | --- |
| Somatic Symptoms and Functional Impairments Caused by the Disease | Qualitative research -phenomenological, grounded theory, descriptive - High | Downgrade one level Moderate* | Remains unchanged | Moderate** | The findings came from 10 papers |
|  |  |  |  |  | *Downgraded one level as the majority of studies (9 out of 10) scored 3 on questions related to the appropriateness of the conduct of the study |
|  |  |  |  |  | **Remains unchanged as all findings unequivocal |
| Multidimensional Impacts on Daily Life | Qualitative research-phenomenological, grounded theory, descriptive - High | Downgrade one level Moderate* | Remains unchanged | Moderate | The findings came from 10 papers |
|  |  |  |  |  | *Downgraded one level as the majority of studies (9 out of 10) scored 3 on questions related to the appropriateness of the conduct of the study |
|  |  |  |  |  | **Remains unchanged as all findings unequivocal |
| Impacts on Mental Health | Qualitative research-phenomenological, grounded theory, descriptive - High | Downgrade one level Moderate* | Remains unchanged | Moderate | The findings came from 9 papers |
|  |  |  |  |  | *Downgraded one level as the majority of studies (8 out of 9) scored 3 on questions related to the appropriateness of the conduct of the study |
|  |  |  |  |  | **Remains unchanged as all findings unequivocal |
| Navigating the Journey of DPN Management | Qualitative research-phenomenological, grounded theory, descriptive - High | Downgrade one level Moderate* | Remains unchanged | Moderate | The findings came from 7 papers |
|  |  |  |  |  | *Downgraded one level as the majority of studies (6 out of 7) scored 3 on questions related to the appropriateness of the conduct of the study |
|  |  |  |  |  | **Remains unchanged as all findings unequivocal |
